# All‐Flex Plasma Patch for In Vivo Delivery of Reactive Species

**DOI:** 10.1002/advs.76049

**Published:** 2026-06-11

**Authors:** Luxiang Zhao, Abulaihaiti Tuergong, Hanshu Yang, Shuang Xue, An Yan, Mingzhen Wang, Weilong Song, Wenzhe Si, Ruixue Wang

**Affiliations:** ^1^ College of Mechanical and Electrical Engineering Beijing University of Chemical Technology Chaoyang Beijing China; ^2^ Department of Laboratory Medicine, State Key Laboratory of Vascular Homeostasis and Remodeling, Key Laboratory of Cardiovascular Molecular Biology and Regulatory Peptides, National Health Commission Peking University Third Hospital Beijing China

**Keywords:** cancer research, *in vivo*, pathology, plasma, reactive oxygen species

## Abstract

Wearable cold atmospheric plasma (CAP) systems hold promise for oncology, but clinical translation has been hindered by limited flexibility, suboptimal skin contact, and insufficient mechanistic evidence. Here, we report an All‐Flex Plasma Patch (AFPP), a fully stretchable CAP platform capable of ambient‐air operation, designed for non‐invasive, safe, and effective cancer therapy. In a subcutaneous melanoma model, one‐week AFPP treatment drives pronounced tumor regression, achieving only 1/19 of the tumor volume observed in control group. The device demonstrates enhanced transdermal penetration of reactive oxygen and nitrogen species (RONS) compared with rigid plasma sources, indicating its potential as a cross‐barrier delivery system with excellent biocompatibility and minimal systemic burden. Moreover, we identify adverse responses under excessive exposure and outline an initial therapeutic range balancing efficacy and potential side effects, highlighting its low side effects under optimized conditions. Utilizing this platform, we systematically reveal that AFPP treatment regulates multiple previously unreported key proteins, drives metabolic reprogramming and disrupts calcium homeostasis, and it inhibits tumor progression by orchestrating multiple cell death modalities. This work provides preliminary mechanistic insights supporting the development of safe and effective wearable CAP therapy, and outlines a framework relevant to the translational advancement of non‐invasive plasma oncology as a complement to conventional treatments.

## Graphical abstract

A fully flexible plasma patch enables stable, conformal treatment on complex biological surfaces and enhances transdermal delivery of reactive species. This platform achieves significant tumor suppression in vivo and reveals coordinated regulation of calcium signaling, metabolism, and programmed cell death, providing a promising strategy for safe and wearable plasma‐based cancer therapy.

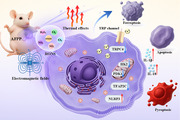

## 1 Introduction

Melanoma is the most aggressive and lethal form of skin cancer. According to the 2025 US cancer statistics, melanoma is expected to account for more than 105,000 new cases and over 8,400 deaths, far surpassing other skin cancers and highlighting its devastating impact on public health [1, 2]. Clinically, melanoma is characterized by early metastatic dissemination, pronounced resistance to conventional radiotherapy and chemotherapy, and dismal prognosis, rendering it one of the most formidable cancers in oncology worldwide [3, 4]. Although the advent of immune checkpoint inhibitors and targeted therapies has markedly improved survival in subsets of patients, their impact is limited by rapid onset of resistance, severe immune‐related toxicities, and prohibitive costs [5]. Consequently, there is an urgent need for novel therapeutic strategies that combine efficacy with safety, convenience, and affordability, ideally through non‐invasive approaches with reduced systemic side effects to meet both scientific and clinical demands.

Cold atmospheric plasma (CAP), enriched with a complex mixture of RONS, electric fields, and ultraviolet radiation [6], has recently been shown to suppress multiple tumor types, including melanoma, in vitro and in vivo [7]. Existing studies show that CAP can induce oxidative stress, DNA damage, and mitochondrial dysfunction, ultimately leading to apoptosis, and may also modulate antitumor immune responses through reactive nitrogen species (RNS)‐mediated pathways [8]. However, most investigations have focused on individual molecular cascades of limited biological significance. As a result, the overall mechanistic basis of CAP therapy in melanoma remains poorly defined and may not fully explain the breadth of its observed therapeutic effects. At the same time, reactive oxygen species (ROS), which play a central role in CAP's anticancer activity, are largely blocked by the skin barrier and cannot be effectively delivered by conventional plasma jets [9]. This transport limitation has fueled controversy regarding the in vivo mechanisms of CAP and further restricted the realization of its full therapeutic potential, underscoring the importance of developing cross‐barrier delivery strategies.

Clinically, melanoma lesions can occur on any skin surface, including anatomically challenging sites with complex morphology and frequent movement [10], yet existing CAP sources face significant structural and material limitations. CAP devices are generally categorized into gap‐type, jet‐type, and surface‐type configurations. Gap‐type plasmas require narrow spacing (≤ 4 mm) to sustain uniform glow, but this inherently limits the treatment area as larger gaps trigger filamentary discharge [11, 12]. Jet‐type plasmas provide relatively stable discharges but are constrained by a small beam footprint and concentrated energy at the tip. Even when arranged in arrays to expand coverage, mutual interference among jets compromises overall uniformity [13, 14]. Surface‐type devices, such as floating‐electrode dielectric barrier discharge (FE‐DBD) [15] and surface dielectric barrier discharge (SDBD) [16], provide broader treatment areas and improved skin contact, making them more suitable for topical applications. Yet even these seemingly promising surface‐type systems are fundamentally constrained by their structural and material choices.

Surface‐type devices typically rely on rigid polymer dielectrics (e.g., polyethylene terephthalate (PET), polyimide (PI), or polytetrafluoroethylene (PTFE)), metallic films, meshes, semiconductor coatings, or conductive inks as electrodes. Such materials inherently lack stretchability and elastic recovery, making it difficult to maintain stable conformal contact on complex physiological surfaces such as joints or dynamic regions [17, 18, 19, 20, 21, 22, 23]. In addition, poor intrinsic biocompatibility raises safety concerns during direct skin contact, posing another barrier to clinical translation. Therefore, despite the promising potential of CAP in oncology, its transdermal efficiency and mechanisms remain insufficiently validated, and no wearable platform integrating flexibility, adaptability, biocompatibility, and safety has been realized.

To address the unmet need for safe and effective plasma application on complex biological surfaces, here we introduce the All‐Flex Plasma Patch (AFPP), a fully flexible and wearable CAP device capable of ambient‐air operation. AFPP combines a polydimethylsiloxane—medical ionic conductive hydrogel—polydimethylsiloxane trilayer with engineered microstructures, achieving stretchability, shape‐memory, and conformal contact while ensuring stable, low‐temperature plasma generation. This design supports cross‐barrier delivery of both ROS and RNS and may help address limitations observed in conventional plasma jets. Through proteomic analysis with experimental validation, AFPP further reveals regulatory entry points in calcium signaling, metabolic control, and inflammatory pathways. Together, these advances highlight AFPP as a route toward non‐invasive, cross‐barrier plasma oncology with both engineering innovation and mechanistic depth.

## 2 Results and Discussion

### 2.1 Discharge Uniformity Control of AFPP via Microstructure Design

In conventional FE‐DBD systems, rectangular or centimeter‐scale gaps often generate overly uniform electric fields or promote long‐range electron migration, leading to rapid electron avalanches that evolve into filamentary discharges. This process produces localized hot spots and non‐uniform plasma patterns, thereby limiting large‐area applications on the human body. To overcome this limitation, we introduced spatially graded microstructures on the PDMS surface to disrupt field uniformity and reshape the local electric field distribution. This strategy effectively suppressed filamentary discharges, improved discharge uniformity, stability, and effective treatment area, and simultaneously lowered the breakdown voltage.

The uniformity of discharge is fundamentally governed by the regulation of plasma density *n*. According to Townsend's theory [24],

(1) $$n=n_{0}\exp\left(Ap\exp\left(\left(-\frac{Bp}{E}\right)d\right)\right)$$

where *n*_0_ is the initial electron density, *E* is the electric field intensity, *d* is the discharge gap, *p* is the pressure, and A and B are constants determined by gas species and external conditions.

**FIGURE 1:**
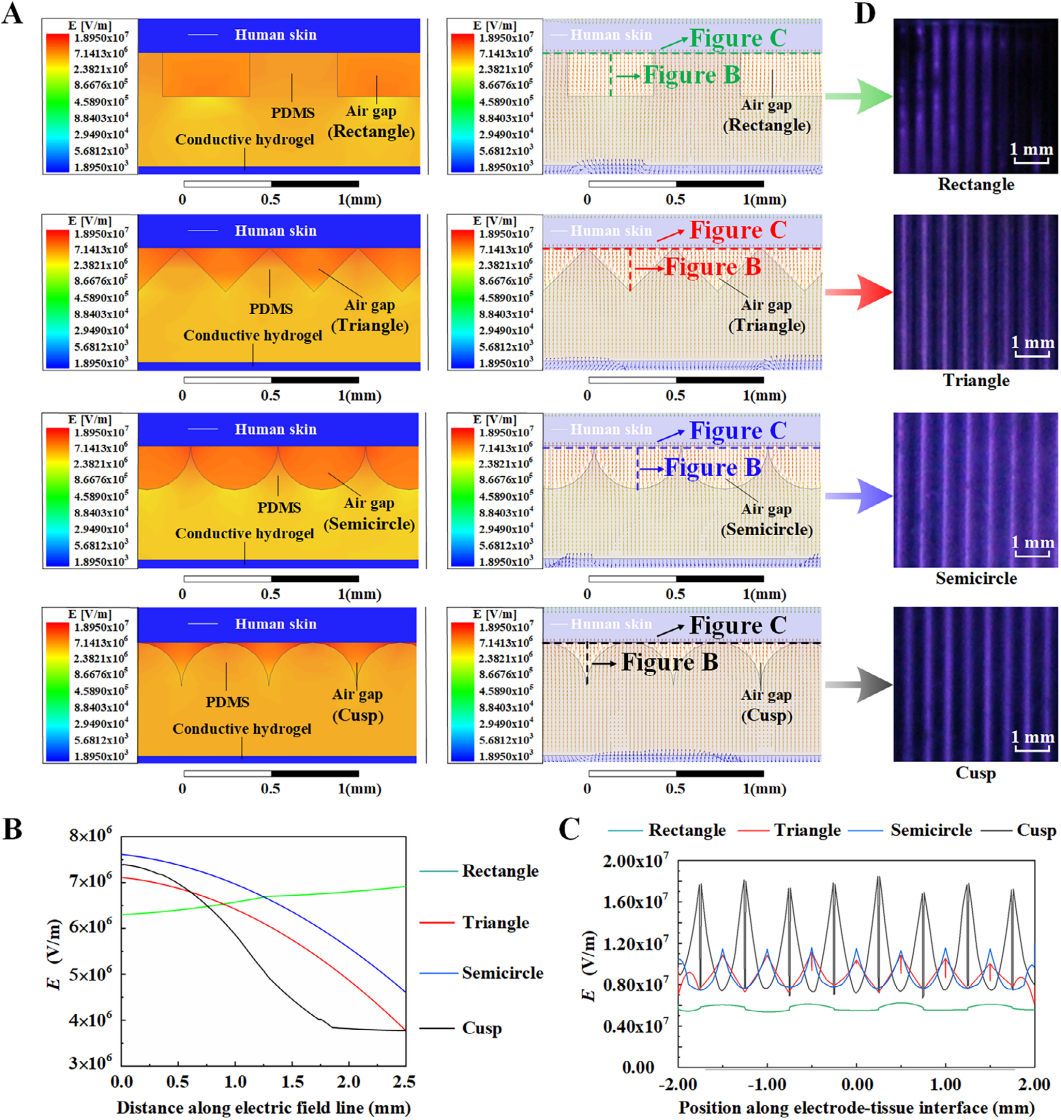
Comparison of electric field distribution and discharge characteristics of AFPP with different surface microstructures. (A) Finite element simulations of electric field distribution and corresponding vector maps, comparing rectangular, triangular, semicircular, and cusp structures within the air gap. (B) Electric field intensity profiles along field lines under different microstructures (indicated by dashed line Figure B in A). (C) Electric field intensity at the electrode‐tissue interface under different microstructures (indicated by dashed line Figure C in A). (D) Representative discharge images obtained under identical experimental conditions for each microstructure.

Therefore, *n* is essentially determined by *n*_0_, *E*, and *d*. When the maximum gap distance (*d*_max_) is excessively large, electron avalanches readily evolve into filamentary discharges. When *d*_max_ is too small, the sharp attenuation of *n*_0_ causes a steep rise in the ignition voltage and increases the risk of breakdown. A microchannel scale of d_max_ = 250 µm was adopted as a practical design parameter to enable controlled avalanche expansion while ensuring discharge stability and safety. Moreover, constructing spatially graded, non‐uniform fields effectively suppressed excessive avalanche development and enhanced discharge diffusion. To this end, we designed four types of PDMS microstructures‐rectangular, triangular, semicircular, and cusp grooves‐and analyzed their electric field distribution via finite element simulations (Figure 1A).

The results showed that the rectangular micro‐grooves, owing to their nearly uniform electric field distribution, produced an almost constant Townsend ionization coefficient, leading to an exponential growth of electron density along the electric field lines within the microcavity (Figure 1B), which strongly favors filamentary discharge. In contrast, triangular, semicircular, and cusp structures exhibited gradually decaying electric field intensity along the field vectors, a pattern that effectively suppressed avalanche overgrowth and improved discharge uniformity [25, 26]. Simulation results along the direction of the electrode‐skin interface (skin surface) (Figure 1C) further revealed that the rectangular structures exhibited a quasi‐uniform surface electric field distribution (E < 0.6 × 10^7^ V/m), whereas the three non‐uniform structures generated surface field intensities exceeding 0.8 × 10^7^ V/m (with local peaks >1.0 × 10^7^ V/m). These strong‐field regions fully covered the skin surface, thereby reducing breakdown voltage and enhancing discharge safety compared with the rectangular configuration. Specifically, the high‐intensity zones at the PDMS‐skin contact points initiated discharge, which then propagated outward into lower‐intensity, longer‐path regions, providing *n*_0_ to sustain plasma expansion. Meanwhile, the peripheral low‐intensity fields delayed electron density growth and further enhanced discharge diffusion.

However, cusp grooves exhibited a maximum field difference approaching 1.0 × 10^7^ V/m, which predisposed them to local dielectric breakdown. In addition, microstructure design must preserve sufficient discharge space within the limited dimensions to maintain adequate seed electron and neutral particle density. The discharge space of cusp grooves was too narrow to maintain a stable plasma. Considering key factors including discharge volume, field distribution, uniformity, safety, and treatment area (as summarized in Table S1), the semicircular groove design demonstrated superior performance and was chosen as the optimal design. Experimental observations (Figure 1D) were highly consistent with the simulations. Under identical applied voltages, rectangular grooves produced uneven discharge filaments, and the large supporting structures significantly reduced the effective treatment area, creating blind zones. Triangular and cusp grooves triggered discharges locally at sharp tips but yielded limited overall coverage and poor uniformity. By contrast, semicircular grooves formed a continuous radial gradient in the discharge channel, effectively guiding electron avalanche expansion. This structure generated a broad and uniform discharge region without filamentary discharge or breakdown events. Its superior performance across all metrics, including uniformity, safety, and effective treatment area coverage, established it as the optimal design for the AFPP.

### 2.2 Spatiotemporal Evolution Simulation of Discharge and Reactive Species Transport in Semicircular‐Microstructured AFPP

**FIGURE 2:**
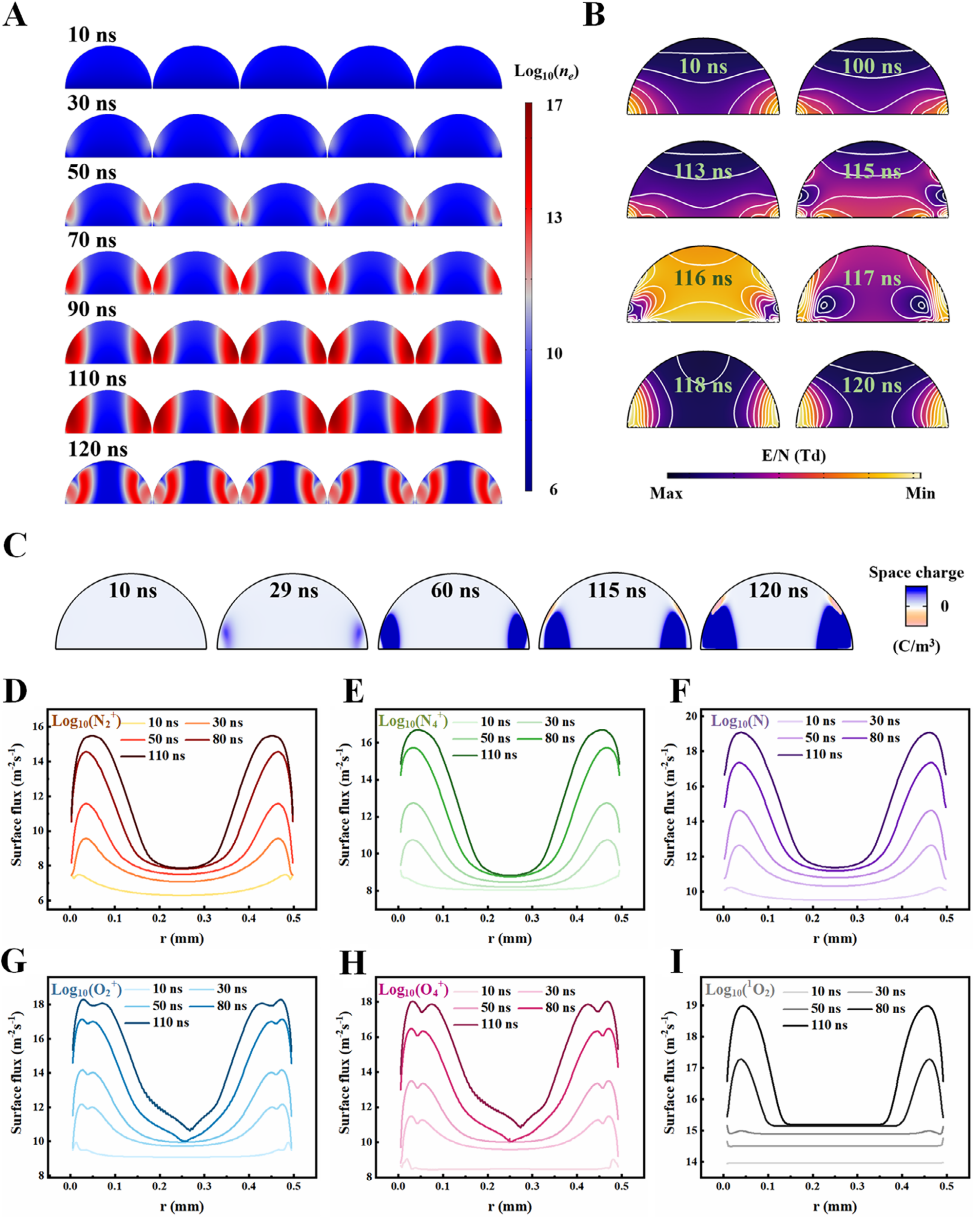
Spatiotemporal evolution of discharge and reactive species in semicircular AFPP under a single‐pulse driving voltage. (A) Temporal evolution of electron density (*n_e_*). (B) Evolution of the reduced electric field (*E/N*). (C) Distribution of space charges during the discharge process. (D) to (I) Spatiotemporal distributions of major reactive species: N_2_^+^, N_4_^+^, N, O_2_^+^, O_4_^+^, and singlet oxygen (^1^O_2_).

To elucidate the discharge process and the flux distribution characteristics of reactive species within the microcavities of the semicircular‐structured AFPP, we conducted spatiotemporal evolution simulations for qualitative analysis (Figure 2). A single‐pulse voltage waveform with a peak amplitude of 3.5 kV, rise/fall time of 10 ns, and pulse width of 100 ns was used in place of the alternating waveform applied in experiments to reduce computational cost. Previous studies have demonstrated that this approach can qualitatively reproduce the discharge events occurring within one AC cycle [27, 28].

At 10 ns, no visible discharge was observed, but the applied positive voltage ionized electrons within the micropores, generating seed electrons for subsequent breakdown. By 30 ns, plasma ignition began at the edges of the semicircular structures, where local electron density sharply increased. Continued voltage application intensified discharges within the gaps, and progressive ionization raised the electron density to ≈10^17^ m^−^^3^ at 110 ns. During the voltage fall stage (Movie S1), the electron density distribution exhibited an intriguing shift, expanding radially toward the center of the micropores. At 120 ns, influenced by residual charges and the reverse electric field between electrodes, weak electron ionization reappeared near the electrode edges.

The electric field in plasma consists of both the externally applied electrostatic field and the self‐consistent field generated within the plasma. The evolution of plasma discharge is therefore closely tied to variations in the self‐built field. Accordingly, the temporal dynamics of the reduced electric field (*E/N*) are commonly used to interpret discharge morphologies (Figure 2B). During the voltage rise (0–10 ns) and plateau (10–110 ns) stages, the overall distribution of *E/N* showed no major changes. The onset and development of discharge expanded the high‐intensity regions while compressing the equipotential lines radially toward the micropore center, resulting in local bending of the potential contours. In contrast, during the voltage fall stage, *E/N* exhibited complex evolution. The high‐intensity regions slid inward from both ends of the micropores toward the radial center, where an extremely low *E/N* zone emerged at the initial discharge site. We speculate that this may result from the reverse electric field generated during the voltage fall, which induced an accumulation of negative charges in this region. Excess charge buildup, under conditions of abundant seed electrons, caused secondary ionization of the gas, thereby giving rise to a new high‐intensity zone. Toward the end of the voltage fall, the *E/N* distribution returned to a pattern resembling that of the rise and plateau stages. Notably, however, the high‐intensity region exhibited a more pronounced axial extension compared with earlier phases. The motion of electrons as well as positive and negative ions under the electric field is a critical factor in the formation of the plasma self‐consistent field. During one voltage cycle, dynamic variations in space charge distribution within the micro/nanopores strongly influenced the evolution of the self‐consistent field (Figure 2C). At 10 ns, corresponding to the pre‐ionization stage, positive and negative charges were balanced and no distinct distribution was observed. By 29 ns, the accumulation of positive space charges at the pore edges indicated the initiation of plasma discharge at these locations. As the discharge progressed, the density of positive space charges increased and extended radially toward the pore center, driving the discharge front inward. During the voltage fall stage, negative space charge accumulation appeared at the axial mid‐height near the pore edges and gradually shifted upward with time, providing evidence for secondary ionization of the gas during this phase.

Because reactive species mediate the antitumor effects of plasma [29], we analyzed their spatiotemporal distributions (Figures 2D–I). On the skin‐contacting surface, most species displayed a bimodal pattern, likely arising from steep potential gradients at the electrode‐skin interface. With discharge progression, fluxes increased and all distribution peaks shifted radially inward, promoting more uniform delivery across the surface. For N_2_^+^ (Figure 2D), the peaks appeared at approximately 0.05 mm and 0.45 mm, with a maximum flux of ≈3.1 × 10^15^ m^−2^s^−1^. N_4_^+^, a short‐lived species generally difficult to detect experimentally, displayed a distribution similar to N_2_^+^ (Figure 2E), with a maximum flux of ≈1.9 × 10^16^ m^−2^s^−1^. Compared with these nitrogen ions, atomic nitrogen (N) exhibited much higher surface fluxes, peaking at ≈1.2 × 10^19^ m^−^^2^ s^−^^1^. Oxygen, the other major component of air, also contributed abundant ionized and excited species during treatment. Notably, O_2_^+^ and O_4_^+^ displayed broader radial distributions in high‐flux regions than their nitrogen counterparts (Figure 2G, H), with peak fluxes of ≈1.1 × 10^18^ m^−2^s^−1^ and ≈9.8 × 10^17^ m^−2^s^−1^, respectively, which is beneficial for achieving uniform plasma treatment and homogeneous reactive species delivery. Among all reactive species, singlet oxygen (^1^O_2_) is particularly critical in plasma‐based cancer therapy. In the flexible micro/nanostructured FE‐DBD used in this study, ^1^O_2_ emerged as one of the dominant species, reaching a high flux of ≈9.9 × 10^18^ m^−2^s^−1^ on the treated skin surface. Although the computational model could not encompass all possible reactive species due to cost limitations, classical reaction pathways leading to their generation allowed further inference (Text S1).

Collectively, spatiotemporal simulations revealed that semicircular microstructures promote stable, diffuse discharges at low voltages and sustain high‐flux, uniformly distributed RONS at the skin interface. Given the established roles of these species in apoptosis, oxidative stress, and metabolic reprogramming, these results provide preliminary mechanistic evidence that semicircular‐microstructured AFPP can generate a broad spectrum of biologically relevant RONS under ambient air, thereby supporting subsequent transdermal delivery and therapeutic efficacy.

### 2.3 Discharge Performance and Electrical Response Regulation of AFPP

**FIGURE 3:**
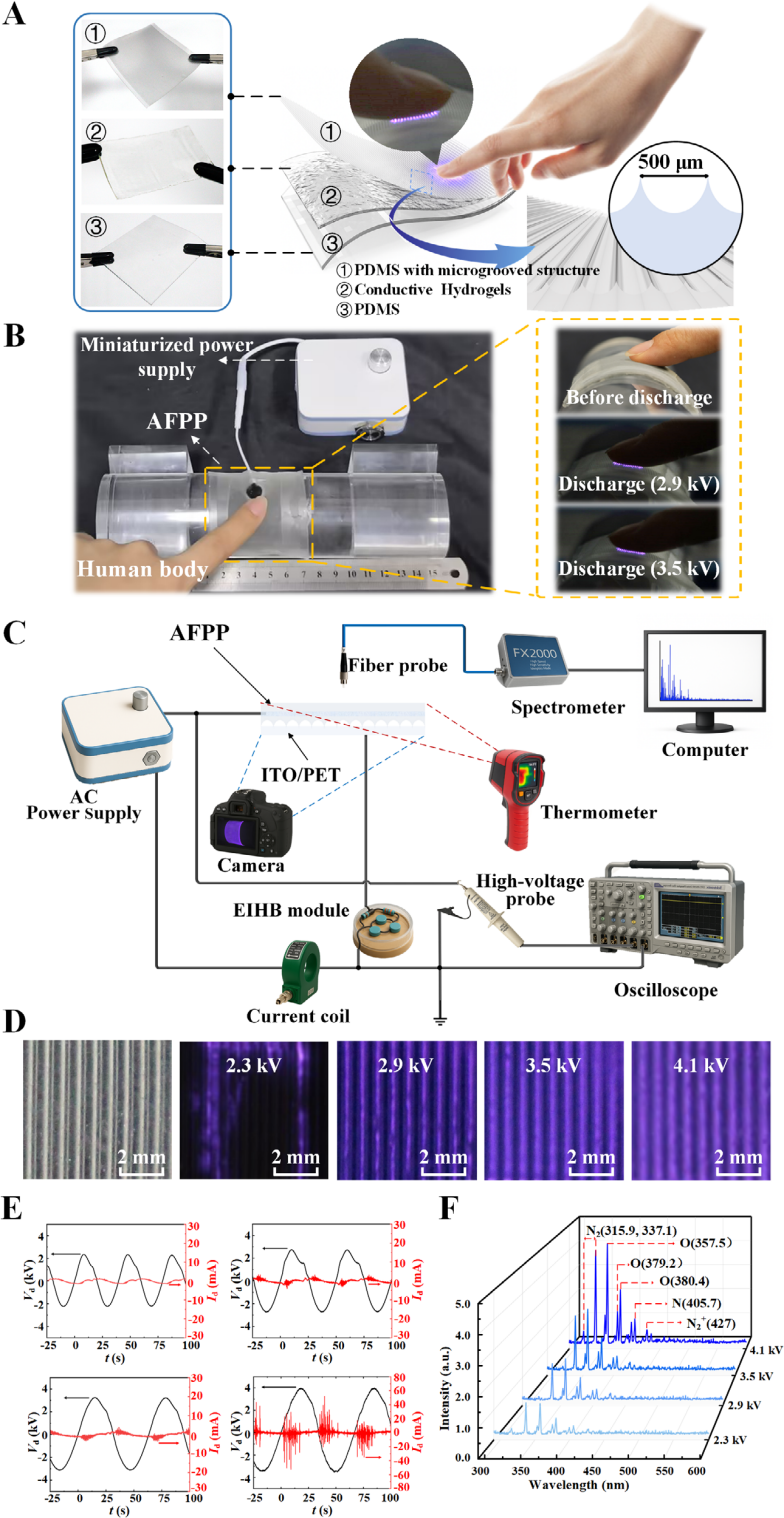
Discharge behavior and electrical performance of AFPP. (A) Schematic of AFPP structure. (B) Photograph of the integrated system and skin‐mimicking test platform. (C) Schematic of the multi‐parameter diagnostic setup. (D) Discharge images under driving voltages of 2.3–4.1 kV. (E) Current‐voltage waveforms at different voltages. (F) Optical emission spectra, with characteristic peaks assigned to N_2_, O, and N_2_^+^ species.

To enable safe and reproducible skin‐conformal discharge, we integrated AFPP with a miniaturized high‐frequency power supply and systematically evaluated its electrical characteristics across representative voltages (Figure 3).

The device adopts a PDMS‐conductive hydrogel‐PDMS trilayer, where microgrooved PDMS dielectrics improve skin conformity and localized electric control. The compact AC power supply featured an outer volume of ≈ 86.4 cm^3^ (effective internal volume 66.9 cm^3^), fulfilling portability requirements (Figure 3A, B).

Based on the IEC 60990 human‐equivalent circuit model (Figure 3C), we established a multi‐parameter diagnostic platform enabling synchronous monitoring of optical emission spectra, current‐voltage characteristics, and thermal responses. The results revealed that discharge was initiated at 2.3 kV, where only weak local plasmas were observed. At 2.9–3.5 kV, AFPP entered a uniform glow‐like mode covering nearly the entire electrode surface (Figure 3D). The corresponding current‐voltage waveforms exhibited typical dielectric barrier discharge (DBD) features, with current peaks below 5 mA, well under the commonly accepted medical safety threshold of 10 mA, indicating a favorable margin of electrical safety under conformal application. At 4.1 kV, instantaneous pulses up to 52 mA indicated energy overload and unstable discharge (Figure 3E).

Lissajous figures further corroborated these findings (Figure S1): at 2.9 and 3.5 kV, the voltage‐charge trajectories formed stable closed ellipses, indicative of reversible charge accumulation‐release cycles under stable energy input, whereas at 4.1 kV the distorted trajectory reflected excessive energy deposition and unstable discharge. With an effective discharge area of 1 cm^2^, the average power density was 0.086 W/cm^2^ at 2.9 kV and 0.111 W/cm^2^ at 3.5 kV, underscoring the low‐power nature of the system, which can be continuously driven by a single rechargeable battery. This power level also renders the AFPP compatible for coupling with energy‐harvesting modules such as triboelectric or photovoltaic systems [30].

Optical emission spectroscopy (OES) was employed to systematically characterize the RONS generated during the actual discharge process of the entire AFPP device (Figure 3F). Distinct emission peaks corresponding to O radicals (357.5 nm, 379.2 nm), the N_2_ second positive system (315.9 nm, 337.1 nm, etc.), and N_2_^+^ (427 nm) [31] were clearly identified. Spectral intensities of these peaks increased progressively as the applied voltage rose from 2.3 to 4.1 kV. Importantly, excited nitrogen molecules can react with ambient oxygen to yield secondary species such as OH and NO radicals [32, 33], which were subsequently confirmed in the liquid phase (Figure 5F). These species have been widely reported to induce tumor cell apoptosis and inhibit tumor progression, thereby playing crucial antitumor roles. Taken together, considering discharge area, uniformity, electrical safety, and RONS generation, we identified 2.9 and 3.5 kV as representative low‐ and high‐voltage operating conditions for subsequent biological experiments.

### 2.4 Mechanical and Operational Stability Assessment of AFPP

**FIGURE 4:**
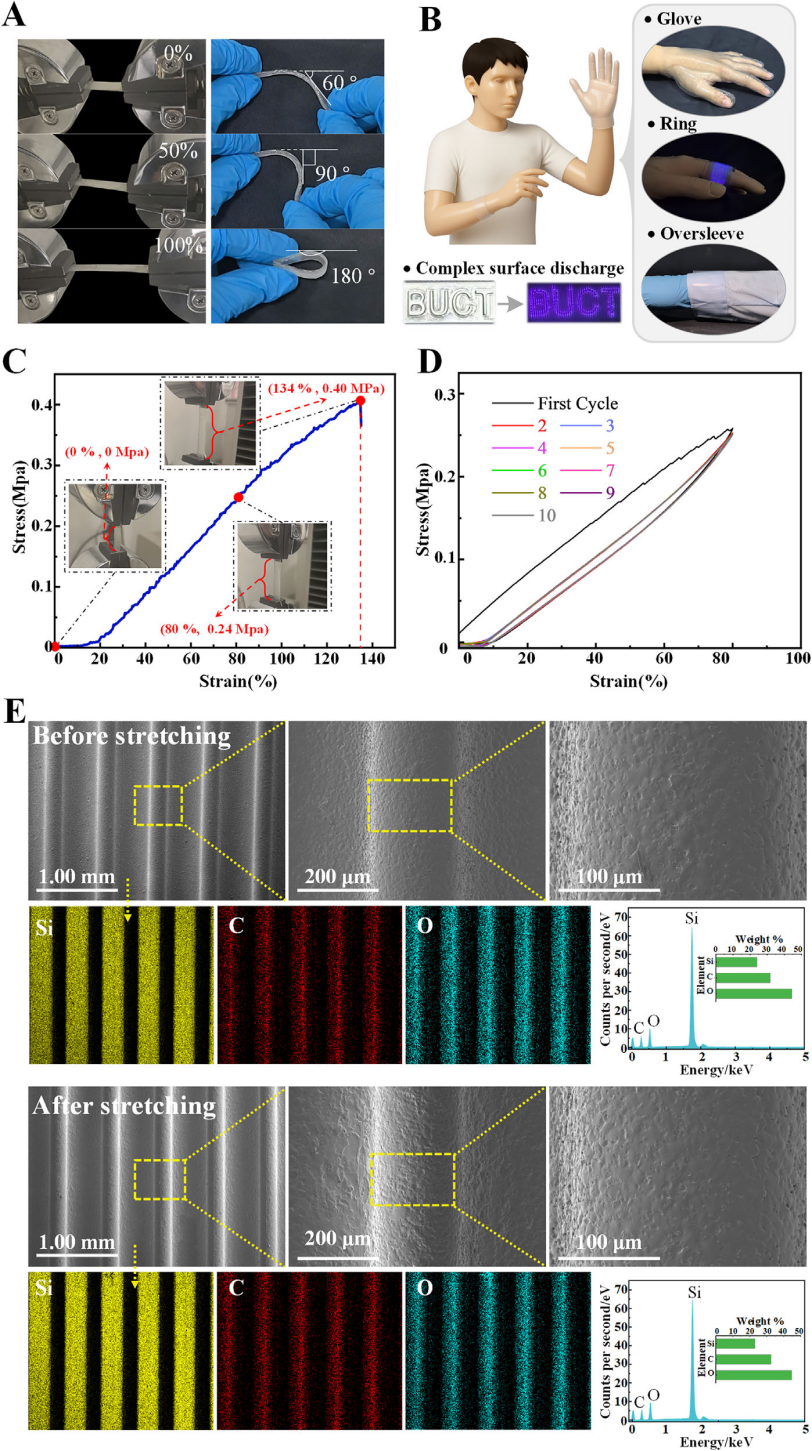
Mechanical deformability and electrical/structural stability of AFPP. (A) Photographs of the patch under uniaxial stretching (0%–100% strain) and multi‐angle bending (60°, 90°, 180°). (B) Discharge demonstration of the patch on irregular human surfaces, including fingertip, forearm, and full‐hand glove, along with the luminescent “BUCT” pattern. (C) Stress‐strain curve at a maximum strain of 134%, with inset images illustrating the deformation process from initial state to fracture. (D) Ten‐cycle tensile test at 80% strain. (E) SEM images and elemental mapping (Si, C, O) of the patch surface before and after 10 stretching cycles.

To ensure long‐term stable and safe therapeutic output of AFPP on complex physiological surfaces and under dynamic conditions, we systematically evaluated its mechanical flexibility, structural durability, and discharge stability (Figure 4).

As shown in Figure 4A, AFPP maintained continuous and intact morphology under uniaxial stretching (0%–100% strain) and multi‐angle bending (60°, 90°, 180°), and rapidly recovered its original shape after load release, demonstrating excellent deformability and resilience. A real‐time manual deformation demonstration, including bending, stretching, twisting, and elastic recovery, is provided in Movie S2, further confirming the flexibility and mechanical robustness of AFPP. Furthermore, in multiple wearing scenarios‐including fingertip, forearm, and full‐hand glove‐the device conformed well to irregular surfaces and maintained stable discharge (Figure 4B). These performances arose from the synergistic effects of the PDMS substrate and conductive hydrogel layer, which together provided both soft conformity and good biocompatibility with biological tissue. Mechanical testing showed that AFPP sustained continuous load response up to 134% strain, with a fracture strength of 0.40 MPa (Figure 4C), reflecting high ductility and tensile strength. All tests followed the ASTM D412 standard method [34], ensuring reliability and comparability. At 80% strain, 10‐cycle tensile tests produced stable hysteresis loops and consistent stress‐strain responses (Figure 4D), indicating strong resistance to mechanical fatigue. Compared with conventional rigid or brittle CAP sources, AFPP represents a fundamental breakthrough in multidimensional mechanical performance.

To evaluate the stability of the AFPP under mechanical deformation and long‐term operating conditions, we systematically tested its mechanical structure, electrical parameters, and surface chemical composition. An LCR meter was used to monitor changes in the device's capacitance following cyclic deformation. The results showed no significant drift in the electrical parameters of the AFPP (Table S2). Concurrently, scanning electron microscopy (SEM) coupled with elemental mapping analysis revealed that the surface microstructure of the AFPP remained intact after cyclic stretching. No cracks or collapses were observed, and the distribution and relative composition of Si, C, and O elements remained largely stable (Figure 4E), demonstrating its excellent mechanical structural stability. To assess material stability and electrical reliability under prolonged plasma exposure, we compared the capacitance variations of the AFPP under continuous discharge, as well as its surface morphology and chemical composition before and after discharging for 6 and 720 min (Figure S2, S3, Table S3). After 6 min, the electrode structure remained intact without significant fluctuations in elemental distribution, indicating that the AFPP does not undergo obvious material oxidation or degradation at a standard single‐treatment dose. However, after 720 min of continuous discharge, the PDMS surface exhibited slight roughening and microcracking, accompanied by a decrease in carbon content (from 46.06% to 30.07%) and an increase in oxygen content (from 31.95% to 47.24%). XPS further revealed that after 720 min, a new C‐O characteristic peak (286.2 eV) emerged in the C 1s spectrum, and the high‐oxidation‐state components in the Si 2p spectrum increased significantly [35]. This suggests that prolonged discharge induces the oxidative cleavage of methyl groups on the PDMS surface and promotes the formation of a silicon‐oxygen network. Nevertheless, the overall capacitance change rate of the AFPP after 720 min of continuous discharge was only 0.356% (Table S3), indicating that these alterations primarily constitute surface oxidative aging and do not significantly compromise the dielectric integrity and electrical stability of the device.

In summary, AFPP exhibits significant advantages in both mechanical and electrical properties. Its high flexibility, fatigue resistance, and interfacial adaptability enable reliable conformal contact with complex physiological surfaces, while its structural integrity and electrical stability under extended discharge cycles ensure consistent and controllable therapeutic output. With this combined mechanical‐electrical robustness, AFPP effectively overcomes the limitations of conventional CAP sources in conformity and stability, providing a reliable foundation for wearable plasma therapy on complex body surfaces.

### 2.5 Antitumor Effects of AFPP in an in Vivo Melanoma Model

**FIGURE 5:**
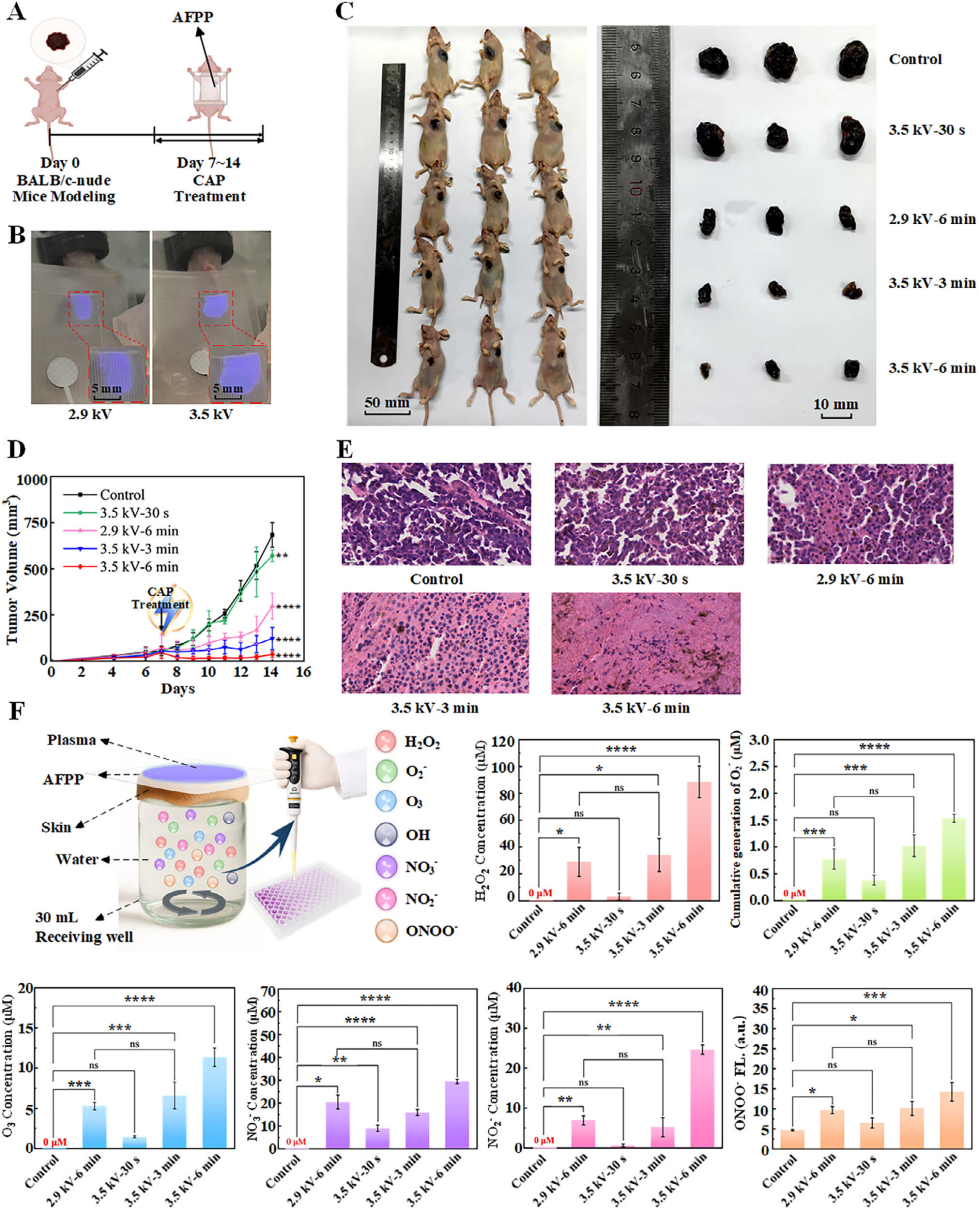
Evaluation of AFPP efficacy in melanoma therapy. (A) Establishment of subcutaneous melanoma in BALB/c nude mice, followed by daily AFPP treatment from day 7 to day 14. (B) Discharge of the flexible patch conformally attached to mouse tumors under 2.9 and 3.5 kV. (C) Representative images of mice (left) and excised tumor tissues (right) from different treatment groups. (D) Tumor volume growth curves for each group. (E) H&E staining of tumor tissues after CAP treatment. (F) Schematic of transdermal collection and measurement of liquid‐phase reactive species, along with the accumulated concentrations of H_2_O_2_, O_2_^−^, O_3_, ·OH, NO_2_^−^, NO_3_^−^, and ONOO^−^ in different treatment groups (unit: µM). Statistical analysis was performed using one‐way ANOVA (^*^ *p* < 0.05, ^**^ *p* < 0.01, ^***^ *p* < 0.001, ^****^ *p* < 0.0001, ns: not significant).

To evaluate the therapeutic efficacy of AFPP in solid tumors, we established a subcutaneous melanoma model in the axilla of BALB/c nude mice (Figure 5A). When the tumor volume reached ≈ 40 mm^3^, AFPP treatment was applied daily for 7 consecutive days under different voltages and durations (2.9 kV‐6 min, 3.5 kV‐30 s, 3.5 kV‐3 min, and 3.5 kV‐6 min) (Figure 5B, C; Movies S3, S4). The results showed a significant reduction in tumor volume compared with the control group, with the 3.5 kV‐3 min and 3.5 kV‐6 min regimens producing the most pronounced tumor inhibition, with no statistical difference between the two groups. In these two groups, tumor growth was markedly suppressed following the initiation of AFPP treatment on day 7, with significant differences emerging from day 9 onward (Figure 5D, P < 0.05). H&E staining further confirmed reduced cellular density and extensive necrotic areas in tumors from the treated groups (Figure 5E), indicating that AFPP exerts significant in vivo antitumor effects under appropriate parameters.

To explore the potential mechanisms underlying the antitumor activity of AFPP, we constructed a liquid‐phase transdermal delivery model to simulate the transdermal transport process of various reactive species under AFPP treatment (Figure 5F). Due to its high structural and functional similarity to murine skin and its common application in transdermal drug delivery and membrane kinetics studies [36], chicken skin with the subcutaneous fat layer removed was utilized as a barrier material to simulate the barrier effect of biological skin. The AFPP was attached directly to the outer surface of the chicken skin for treatment. Immediately following the treatment, the receiving water beneath the barrier was collected, and the concentrations of H_2_O_2_, O_2_^−^, O_3_, ·OH, NO_2_^−^, NO_3_^−^ and ONOO^−^ were quantified. All species except ·OH were detected, and their concentrations increased significantly with higher voltage and longer exposure time, displaying a clear dosage‐dependent trend.

Previous studies reported partial melanoma inhibition by CAP, but mechanisms remain debated, especially regarding in vivo delivery of reactive species [37, 38]. Approximately a decade ago, Szili et al. demonstrated the feasibility of RONS penetrating tissue barriers using plasma jets [39, 40, 41]. Building upon these studies, Duan et al. further indicated that ROS generated by CAP jets are unable to penetrate murine skin (200–300 µm), whereas RNS can penetrate the skin more effectively [9]. By contrast, AFPP enabled trans‐barrier delivery not only of RNS (NO_2_^−^, NO_3_^−^ and ONOO^−^) but also of ROS (H_2_O_2_, O_3_, O_2_^−^), whereas ·OH remained undetectable. Unlike CAP jets, which rarely achieve ROS penetration, this outcome likely arises from the FE‐DBD configuration, where tissue functions as part of the electrode. Such intimate plasma‐tissue coupling promotes charge injection and localized RONS generation at the interface, thereby fundamentally enhancing transport dynamics. Particularly, when discharge occurs in direct contact with tissue, certain ROS may bypass superficial quenching mechanisms and traverse the barrier at effective concentrations.

Mechanistically, even when ROS penetration occurred, RNS still demonstrated higher transdermal delivery efficiency, with concentrations increasing with voltage and exposure duration. This may be linked to the lipophilicity of nitro‐compounds and the nitrosative modifications they induce [42, 43]. Conversely, highly reactive ·OH is rapidly consumed in non‐enzymatic reactions with epidermal proteins and lipids [44, 45]. H_2_O_2_, though longer‐lived, is readily neutralized by antioxidants (e.g., vitamins E/C, carotenoids, glutathione) in the skin [46, 47]. Collectively, these findings challenge the conventional assumption that “ROS cannot penetrate skin” and highlight the critical role of FE‐DBD configurations in reactive species transport and therapeutic responses. This suggests that CAP penetration depends not only on the species generated but also on discharge structure and plasma‐tissue interaction mode.

From a therapeutic mechanism perspective, efficient delivery of RNS‐particularly NO_2_^−^, NO_3_^−^ and ONOO^−^, was strongly correlated with melanoma suppression, suggesting they may play a dominant role in CAP‐induced tumor inhibition [48, 49]. Literature reports indicate that: (1) NO_2_^−^ can convert to cytotoxic NO_x_ species in acidic microenvironments [50, 51], (2) ONOO^−^ disrupts the mitochondrial electron transport chain by inducing protein tyrosine nitration [52], and (3) nitro‐lipid formation can activate death receptor signaling pathways [53]. Among these species, ONOO^−^ possesses both strong oxidative and nitrative capacities. In our experiments, its liquid‐phase concentration followed the same trend as NO_2_^−^ and NO_3_^−^, and its transdermal permeability was highly consistent with prior simulation studies [54, 55]. It can traverse phospholipid bilayers via passive diffusion [56], providing a clear physical basis for its efficient delivery. More importantly, ONOO^−^ induces nitrosative stress that triggers cell death, a mechanism already established as one of the major pathways for plasma‐mediated tumor cell killing [57].

We further noted that the correlation between RONS levels and tumor suppression was not strictly linear. For instance, RONS levels in the 2.9 kV‐6 min and 3.5 kV‐3 min groups showed no significant difference, yet the latter achieved stronger tumor inhibition. This “dosage‐effect deviation” suggests that beyond RONS‐driven chemical effects, discharge‐induced physical mechanisms may also contribute. Infrared thermography revealed that under 3.5 kV, the tumor surface temperature increased from 37.4°C to 47.7°C within 1 min. Previous studies indicate that such mild hyperthermia does not cause tissue damage but enhances skin permeability and local perfusion [58, 59], thereby may jointly promote RONS delivery and amplify therapeutic effects.

In summary, AFPP demonstrated strong antitumor potential in vivo. Its therapeutic mechanisms involve not only deep chemical delivery of RONS but also discharge‐induced physical co‐factors such as mild hyperthermia. The observation that treatment efficacy varied despite similar RONS concentrations suggests that RONS dosage is not the sole determinant. This “physicochemical synergy” underscores the existence of an adjustable therapeutic window: introducing controlled mild hyperthermia under safe conditions may enhance skin permeability, improve RONS delivery efficiency, and further amplify CAP therapeutic effects. This finding challenges the traditional paradigm of minimizing thermal effects in CAP therapy and highlights the need to integrate both RONS concentrations and physical parameters (e.g., electric field intensity, thermal effects) into plasma dosimetry. Such an approach will enable the development of more comprehensive dosage‐response models and facilitate the precise and controllable clinical translation of CAP for solid tumor therapy.

### 2.6 Safety Evaluation of AFPP Cancer Therapy

**FIGURE 6:**
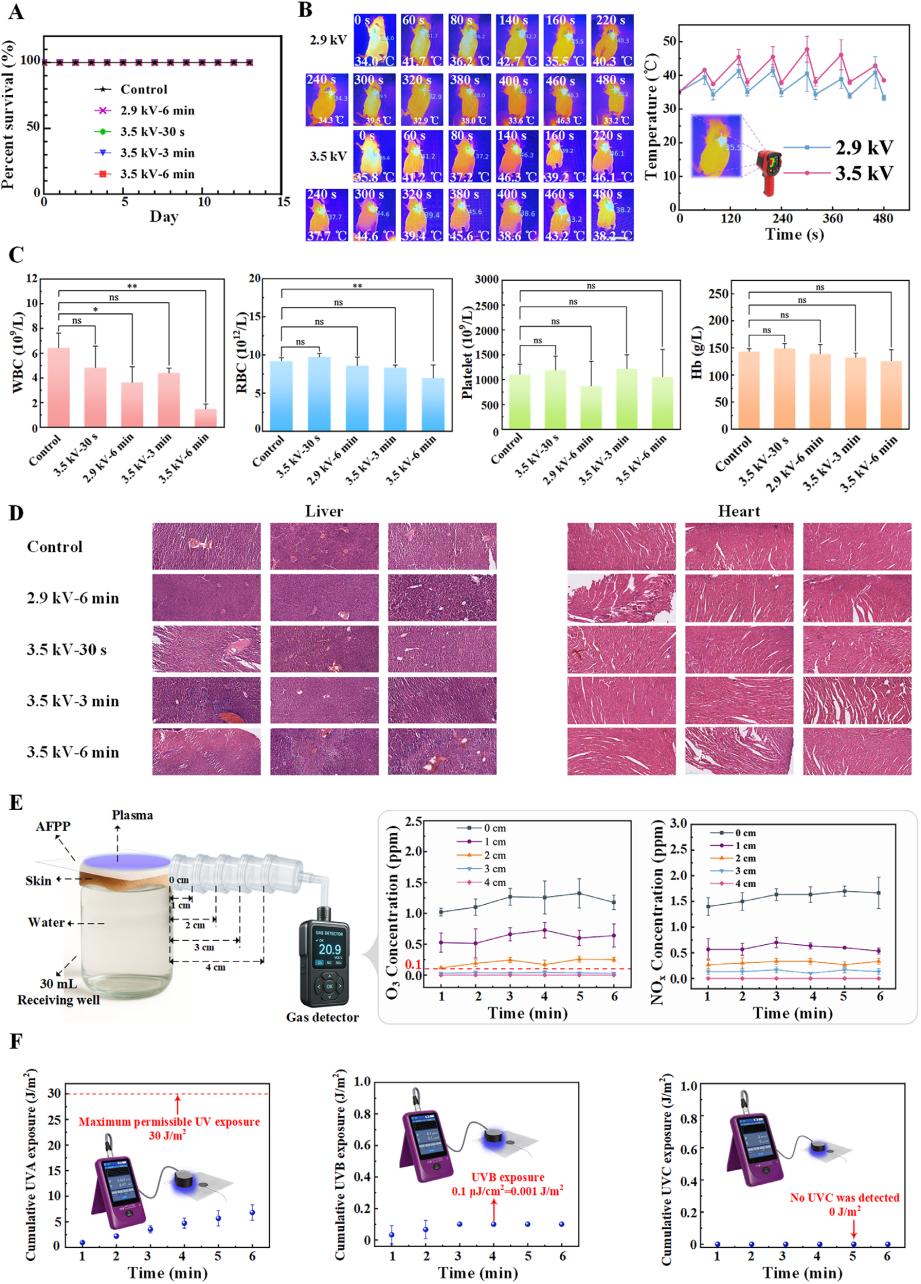
In vivo safety assessment of AFPP melanoma treatment. (A) Survival monitoring of mice over 14 days. (B) Temperature profiles of the treated skin under different discharge conditions, including representative infrared thermographs (left) and temperature evolution curves (right). (C) Hematological parameters, including white blood cells (WBC), red blood cells (RBC), platelets, and hemoglobin (Hb). Data are presented as mean ± SD (n = 6). (D) H&E‐stained histological sections of major organs (liver, heart) from different groups. (E) Monitoring of O_3_ and NO_x_ release during AFPP discharge, including temporal concentration curves (left) and distance‐dependent distributions (right), for evaluating potential exposure risks. (F) UV exposure levels of the AFPP under 3.5 kV‐6 min. During the test, the UV probe was placed in direct contact with the AFPP discharge area to evaluate potential UV risks. Statistical analysis was performed by one‐way ANOVA (^*^ *p* < 0.05, ^**^ *p* < 0.01, ns: not significant).

To systematically evaluate thermal effects, physiological toxicity, and potential environmental risks during AFPP treatment, we performed a comprehensive safety analysis across survival, blood, tissue, thermal, and gas dimensions (Figure 6). As shown in Figure 6A, no mouse deaths occurred in any treatment group during the 14‐day course, with overall survival maintained at 100%, suggesting good physiological tolerance of AFPP in this model. In addition, no statistically significant differences in body weight were observed among all treatment groups throughout the therapeutic period, further indicating the absence of substantial systemic toxicity (Figure S4).

Thermal safety was further assessed by infrared thermography under different parameters (Figure 6B). With an intermittent discharge strategy (60 s on/20 s off), skin temperature at 2.9 kV remained between 33.3–41.4°C, within the biological safety range. At 3.5 kV, transient peaks up to 47.7°C were observed but quickly returned to 37.4–38.5°C during pauses, preventing cumulative heating. No visible skin damage or necrosis occurred under any condition.

Previous studies indicate that protein denaturation and cell death are unlikely when tissue temperature remains below 42°C [60], and even transient elevations above this threshold seldom cause injury. For instance, most normal tissues tolerate up to 44°C for 1 h without damage [61]. In this study, skin temperature in the 2.9 kV group consistently remained below 42°C. Although transient peaks were observed in the 3.5 kV group, the alternating discharge and natural cooling cycles enabled local temperatures to rapidly return to the physiological range, thereby preventing thermal accumulation.

To assess the biological effects of thermal exposure more accurately, we adopted the widely recognized metric of cumulative equivalent minutes at 43°C (CEM43), which enables conversion of exposure times under different temperature conditions into an equivalent dosage, and has been widely used to predict thermally induced injury across various tissue types [60, 62]:

(2) $$\mathrm{CE}M_{43\;}=\Delta tR^{(43-T)}$$

where Δt refers to the total duration of thermal exposure, *T* is the average temperature over a given time interval *t*, and R is assigned a value of 0.25 for temperatures below 43°C and 0.5 for those above.

By this calculation, the 3.5 kV‐6 min condition corresponded to only 4.3 min at 43°C, well below the established thermal damage threshold for skin [60, 62], confirming that intermittent discharge with cooling intervals prevents thermal accumulation and ensures thermal safety.

Systemic effects were further assessed by hematological analysis (Figure 6C). In the high‐dosage group (3.5 kV‐6 min), WBC and RBC levels were significantly reduced (p < 0.01), suggesting stress responses in immune and hematopoietic systems under excessive CAP exposure. The reduction in WBC counts may reflect feedback regulation under oxidative stress rather than direct cytotoxicity [63], whereas reduced RBC counts may arise from RONS‐mediated lipid peroxidation impairing membrane stability and accelerating clearance [64]. These results suggest potential systemic disturbances under high‐dosage CAP exposure, while platelet and hemoglobin levels showed no significant differences among groups.

H&E staining analysis (Figure 6D) revealed coagulative, focal, and patchy necrosis in the liver, and myocardial atrophy in mice of the 3.5 kV‐6 min group, whereas no major damage was detected in other groups. These effects might result from the CAP treatment applied to the surrounding skin region. Although the peak current of the device was controlled below 5 mA, within the limits of biomedical electrical safety standards, the shallow thoracoabdominal cavity and densely arranged organs of nude mice may render the heart and liver more susceptible to localized high‐field regions adjacent to the CAP treatment zone. Even low currents at sustained high frequencies can disrupt cardiomyocyte potentials, calcium balance, and mitochondrial function, leading to metabolic suppression and structural degeneration [65]. On the other hand, RONS are the principal mediators of CAP‐induced biological effects. Their high reactivity enables them to oxidize membrane lipids, mitochondria, and DNA, thereby inducing cellular injury. As the central detoxification organ, the liver is enriched in mitochondria and diverse metabolic enzyme systems, rendering it highly sensitive to oxidative stress. Previous studies have shown that RONS can trigger the opening of the mitochondrial permeability transition (MPT) pore, leading to membrane potential collapse and ATP depletion, and ultimately resulting in oncotic necrosis of hepatocytes [66]. In addition, strong oxidizing species can drive lipid peroxidation, generating highly reactive metabolites such as 4‐HNE and MDA, which further cause protein inactivation, DNA damage, and mitochondrial dysfunction, and are closely associated with cell death [67]. Taken together, the localized hepatic necrosis observed in this study is likely a classical oxidative stress‐induced pathological response elicited by RONS released from CAP.

Under the present experimental conditions, the combined effects of AFPP‐generated RONS and localized electric fields may induce stress responses in organs such as the heart and liver, manifesting as substantive injury in the high‐dose group. These findings suggest that CAP may exert organ‐specific and dose‐dependent adverse effects in small‐animal models through a dual “electric field + RONS” mechanism, underscoring the need to consider deep‐tissue dose parameters and to strengthen cross‐organ safety evaluation in the translational development of AFPP.

To evaluate potential gaseous risks during AFPP discharge, we monitored real‐time concentrations of NO, NO_2_, and O_3_ under different treatment durations and distances (Figure 6E). Even at the maximum discharge intensity and treatment time used in this study, instantaneous concentrations of NO and NO_2_ remained well below the 8 h time‐weighted average (TWA) exposure limits established by the Occupational Safety and Health Administration (OSHA) (NO = 25 ppm, NO_2_ = 5 ppm) [68]. O_3_ concentrations exceeded the safety threshold of 0.1 ppm [69] only within 3 cm of the discharge region, but consistently remained below this limit at conventional operating distances, posing no exposure risk. Furthermore, under natural ventilation and intermittent discharge modes, AFPP‐generated gases were rapidly dispersed, preventing accumulation to harmful levels. Together, the gas concentration profiles, treatment modality, and diffusion characteristics confirm clear gas‐phase safety of AFPP under appropriate operating conditions, laying a solid foundation for its safe implementation in close‐contact clinical applications.

Furthermore, ultraviolet (UV) radiation exposure levels are a key indicator of the safety of plasma devices. We monitored the cumulative UV (230‐400 nm) exposure in the discharge region of the AFPP in real time under the maximum treatment dose condition (3.5 kV‐6 min) (Figure 6F). The results show that the UV radiation generated by the AFPP is primarily concentrated in the UVA (315–400 nm), with a maximum cumulative exposure of 8.479 J/m^2^ after 6 min of continuous discharge, well below the maximum international safety threshold [70] of 30 J/m^2^. Meanwhile, the cumulative exposure of UVB (280–315 nm) was approximately 0.001 J/m^2^, and potentially harmful short‐wave UVC was completely undetected. This characteristic is mainly attributed to the discharge mechanism of air plasma, where high concentrations of N_2_ dominate the excitation and emission processes, concentrating the primary emission signals in the near‐UV and visible regions. Furthermore, UVC radiation (230‐280 nm) is effectively absorbed by molecules such as O_2_ and N_2_ in the discharge gap before reaching the tissue target, ensuring that it does not interact with the treated biological matrix [71, 72]. These test results are consistent with the spectroscopic analysis, further confirming that the UV radiation from the AFPP operating under preset parameters remains within a safe exposure range, posing no risk of phototoxicity or additional radiation damage to the skin.

In summary, AFPP demonstrated overall biosafety under proper parameter control and intermittent discharge. Skin temperature, current, and gas concentrations remained within safety ranges, and only the high‐dose condition (3.5 kV‐6 min) caused mild hematologic changes and stress‐related injury in heart and liver. These results underscore the dose‐dependent nature of systemic toxicity, define an adjustable safety threshold, and provide critical data for clinical translation of AFPP in skin and superficial tumor therapy.

### 2.7 Molecular Mechanisms of AFPP in Melanoma Therapy

**FIGURE 7:**
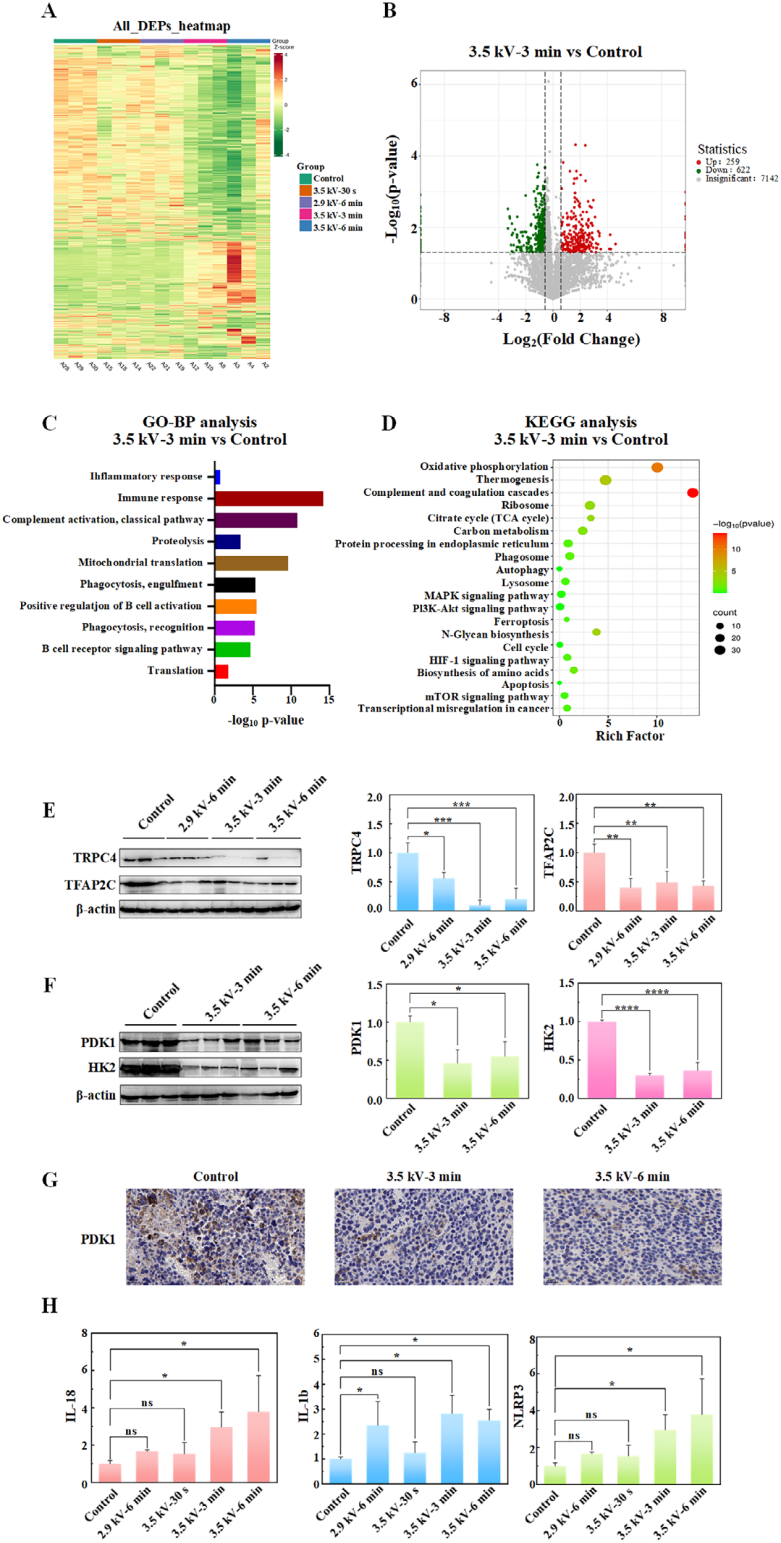
Molecular mechanisms underlying AFPP‐mediated melanoma therapy. (A) Heatmap of protein expression across different treatment groups. (B) Volcano plot of differentially expressed proteins between the 3.5 kV‐3 min group and control. (C) GO enrichment analysis of biological processes involving differentially expressed proteins in the 3.5 kV‐3 min group. (D) KEGG pathway analysis showing the metabolic and signaling pathways enriched in the 3.5 kV‐3 min group. (E) Western blot and quantification of TRPC4 and TFAP2C protein expression. (F) Western blot analysis of PDK1 and HK2 expression across groups. (G) Immunohistochemical staining of PDK1 in melanoma tissues from different treatment groups. (H) mRNA levels of IL‐18, IL‐1β, and NLRP3 across treatment groups. Data are presented as mean ± SD (n = 3). Statistical analysis was performed by one‐way ANOVA (^*^ *p* < 0.05, ^**^ *p* < 0.01, ^***^ *p* < 0.001, ^****^ *p* < 0.0001, ns: not significant).

To dissect the molecular effects of AFPP under different treatment regimens, we performed proteomic profiling of melanoma tissues. Plasma treatment markedly remodeled the tumor proteome (Figure 7A). Under the 3.5 kV‐3 min condition, compared with control group, 259 proteins were upregulated and 622 proteins were downregulated (Figure 7B). GO functional annotation indicated enrichment of biological processes such as immune response, complement activation, and mitochondrial protein synthesis, while KEGG pathway enrichment revealed involvement in oxidative phosphorylation, lysosome, tricarboxylic acid cycle, apoptosis, and autophagy (Figure 7C, D). Extending the treatment duration to 6 min reduced the number of differentially expressed proteins (Figure S5A) and produced a more focused enrichment pattern, highlighting networks associated with lysosome, apoptosis, autophagy, and pyroptosis (Figure S5B, C). These results suggest that AFPP exerts anti‐tumor effects by regulating multiple signaling pathways in a dosage‐dependent manner.

At the molecular level, we observed consistent downregulation of transient receptor potential canonical (TRPC4) and transcription factor AP‐2γ (TFAP2C) across treatment conditions. The proteomics results were further validated by western blotting (Figure 7E), which confirmed a marked reduction, particularly in the high‐voltage groups. TRPC4, a pH‐sensitive member of the transient receptor potential channel family, plays a critical role in regulating calcium homeostasis and microenvironmental adaptation in tumor cells [73]. Given that melanoma cells typically exhibit a reversed pH gradient characterized by extracellular acidification and intracellular alkalinization, our study provides the first evidence that CAP can significantly suppress TRPC4 expression, suggesting that it may inhibit tumor growth by disrupting Ca^2+^ homeostasis.

TFAP2C, an important transcription factor, has been reported to promote malignant progression in multiple cancers, in part by suppressing apoptosis [74], reducing sensitivity to ferroptosis [75], and contributing to therapeutic resistance [76, 77]. In our study, TFAP2C expression was markedly reduced across all treatment groups, indicating that plasma treatment may eliminate this repression mediated by transcription thereby enhancing melanoma sensitivity to apoptosis and ferroptosis and diminishing the potential for drug resistance.

At the metabolic and signaling level, the concurrent downregulation of glycolytic enzymes hexokinase 2 (HK2) and pyruvate dehydrogenase kinase‐1 (PDK1) was particularly distinguished. HK2, the rate‐limiting enzyme of aerobic glycolysis and the only glycolytic enzyme capable of binding to mitochondria, is aberrantly overexpressed in tumors and is considered a hallmark of metabolic reprogramming in cancer cells [78]. Previous studies have shown that HK2 is highly expressed in multiple cancers, where it promotes glucose uptake, lactate production, as well as cell proliferation and angiogenesis [79]. In malignant melanoma, HK2 not only participates in glycolysis [80] but also drives angiogenesis and invasion via activation of the p38‐MAPK pathway [81]. Accordingly, HK2 has been widely recognized as a potential therapeutic target.

PDK1 is a key enzyme implicated in metabolic reprogramming of tumors, is induced in several tumors including glioblastoma, breast cancer and melanoma [82]. In our study, CAP treatment significantly downregulated the protein levels of both HK2 and PDK1 (Figure 7F), and immunohistochemistry further confirmed a pronounced reduction in the expression of PDK1 at the tissue level (Figure 7G). These findings suggest that AFPP suppresses melanoma progression by simultaneously weakening the cellular energy supply and survival signaling, thereby exerting dual‐layered regulation at the levels of metabolism and signal transduction.

In addition to directly suppressing tumor metabolism and survival signaling, we also observed a significant upregulation of mRNA levels of NOD‐like receptor family pyrin domain containing 3 (NLRP3) inflammasome‐related factors, including NLRP3, IL‐1β, and IL‐18, in the treatment groups (Figure 7H), suggesting activation of the inflammasome axis. According to previous studies, the fully assembled NLRP3 inflammasome can activate caspase‐1, thereby inducing gasdermin D‐dependent pyroptosis and promoting the release of IL‐1β and IL‐18, which in turn triggers inflammatory cell death and amplifies immune signaling [83, 84]. Furthermore, as HK2 has been reported to play a role in pyroptosis induction [85], our study further confirmed at the RNA level that CAP treatment significantly increased IL‐1β, IL‐18, and NLRP3 expression in tumor tissues, effectively promoting pyroptosis and potentially activating antitumor immune responses.

Across different treatment regimens, proteomic profiling and molecular validation collectively demonstrated that CAP reshapes the molecular network of melanoma through multiple pathways and layers. Previous reports have shown that CAP can inhibit tumor progression by modulating the tumor microenvironment and influencing programmed cell death [86]. Our study further revealed a multifaceted regulatory landscape in which CAP simultaneously modulates TRPC4 (Ca^2+^ homeostasis), TFAP2C (transcriptional regulation), HK2/PDK1 (metabolism and AKT signaling), and the NLRP3 inflammasome, forming a synergistic network that integrates ion channel regulation, metabolic reprogramming, programmed cell death, and inflammatory immunity. This mechanistic framework not only enriches the molecular basis of CAP as a therapeutic strategy but also provides new evidence and directions for its clinical application in cancer therapy.

## 3 Conclusions

**FIGURE 8:**
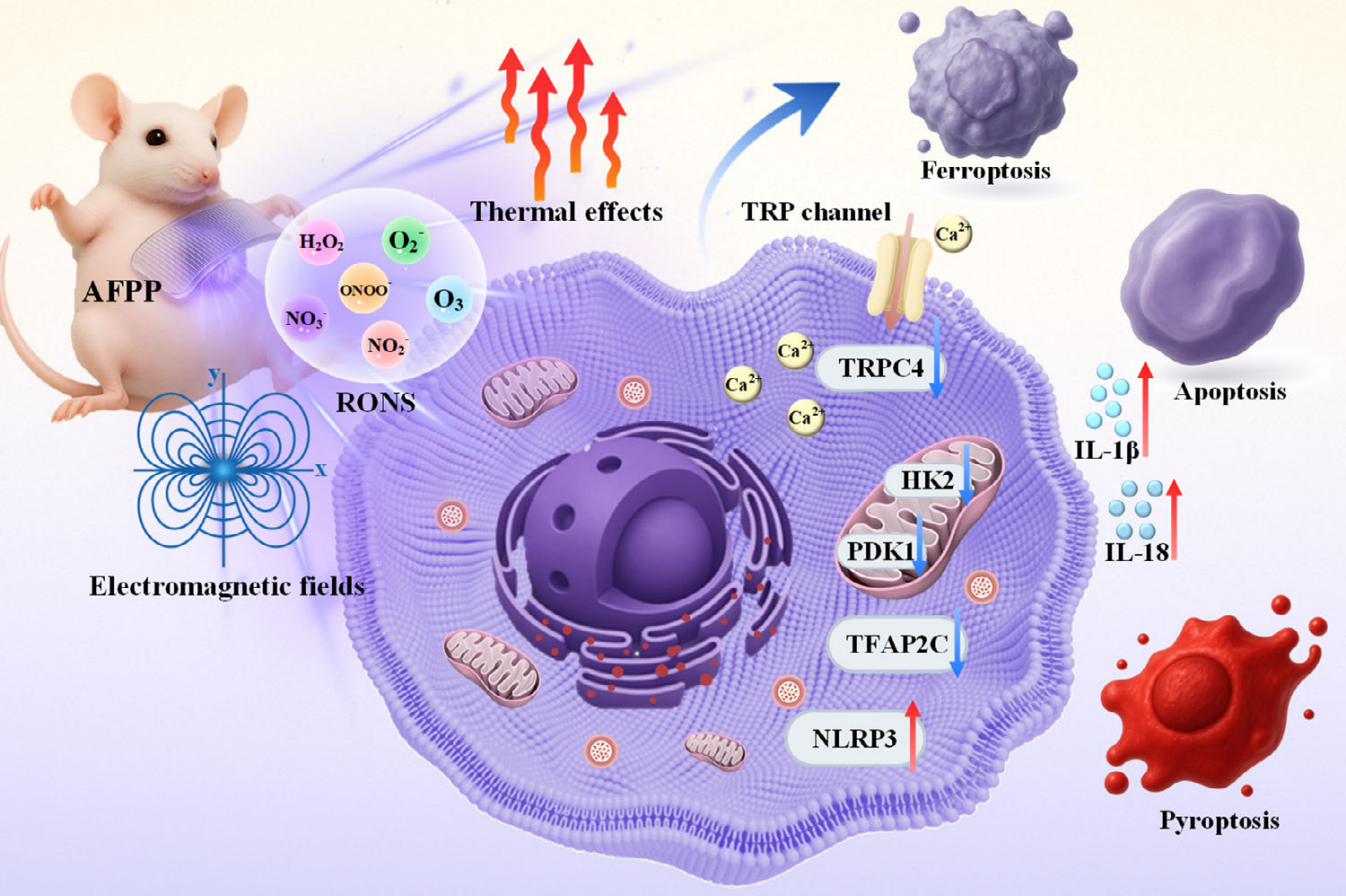
Schematic illustration of the multilayered mechanisms underlying AFPP‐mediated melanoma inhibition. The All‐Flex Plasma Patch (AFPP) enables transdermal delivery of RONS such as H_2_O_2_, O_2_^−^, ONOO^−^, O_3_, NO_3_^−^, and NO_2_^−^, along with mild thermal effects, into tumor tissues, thereby inducing intracellular oxidative stress and signaling alterations. Proteomic and molecular analyses further demonstrate that AFPP coordinately downregulates TRPC4, TFAP2C, HK2, and PDK1, associated with Ca^2+^ imbalance and metabolic changes. Meanwhile, activation of the NLRP3‐caspase‐1 pathway promotes the release of IL‐1β and IL‐18 and triggers pyroptosis, which, together with apoptosis and ferroptosis, suggests a multi‐pathway interaction framework for tumor inhibition.

In this study, we developed and systematically validated an All‐Flex Plasma Patch (AFPP). Unlike previous “flexible CAP” devices that were only bendable to a certain extent, AFPP integrates PDMS with conductive hydrogel to high stretchability, elastic recovery, and stable structural adaptability, thereby enabling seamless conformity to complex and dynamic body surfaces. This design supports stable attachment and uniform plasma exposure in vivo, laying a solid technical foundation for non‐invasive and safe wearable plasma‐based tumor therapy (Figure 8).

Building on this platform, we further elucidated the multilayered mechanisms underlying AFPP‐mediated tumor inhibition. Enhanced transdermal delivery of RONS was primarily attributable to localized thermal effects within the safety threshold, which markedly facilitated the penetration of ROS and thus compensated for the limitation of conventional plasma jets that predominantly deliver RNS. Proteomic profiling further revealed coordinated regulation of several previously unreported molecular targets, including the calcium channel TRPC4, transcription factor TFAP2C, and metabolic regulators HK2 and PDK1. These combined molecular alterations drove metabolic reprogramming and Ca^2+^ disequilibrium, while concurrently activating programmed cell death pathways such as apoptosis and pyroptosis, thereby outlining a multi‐pathway framework for AFPP's antitumor effects. Collectively, these findings establish AFPP as a molecular modulation platform that leverages synergistic electric, thermal, and RONS effects to profoundly reshape cancer cell survival and death trajectories.

Safety assessments further defined the therapeutic window of AFPP. By integrating electrical monitoring, thermal evaluation, and gas analysis with biological readouts, we systematically delineated the safe threshold and effective dose range under different operating conditions. AFPP was biologically well tolerated at optimized voltages and treatment durations, while excessive exposure induced adverse responses. These results suggest that the safety profile of AFPP may depend on a composite of electrical parameters, thermal effects, and RONS exposure, rather than by chemical factors alone, underscoring its low side‐effect profile under optimized conditions.

AFPP opens a new avenue for next‐generation wearable tumor therapy. Compared with conventional chemotherapy or immunotherapy, AFPP offers the advantages of being non‐invasive, cross‐barrier, localized, and cost‐effective, while also mitigating the risk of resistance. Its low‐power requirement further suggests compatibility with triboelectric or photovoltaic energy‐harvesting modules, paving the way toward self‐sustained long‐term therapeutic systems for resource‐limited or emergency settings. Future work should optimize long‐term skin adherence, establish refined dosimetric models, and explore combination strategies with immunotherapy or photothermal therapy. Thus, AFPP not only represents a significant advancement in plasma source design but also provides a safe and paradigm‐shifting platform that bridges plasma physics with precision oncology.

## 4 Experimental Section

### 4.1 Simulation of AFPP

The electrostatic field distributions of AFPPs with different surface microstructures were simulated using Ansoft Maxwell 3D to analyze discharge intensity and uniformity. The discharge process and reactive species flux were further modeled by numerical simulations with COMSOL Multiphysics. The model was established in a Cartesian coordinate system, with a voltage of 3.5 kV applied to the upper electrodes (Figure S6). The overall computational domain was defined as a rectangle with dimensions of 2500 µm × 650 µm, featuring a semicircular microstructure with a radius of 250 µm. The electrode material was modeled as PDMS (relative permittivity *ε*_r_ = 7.5), while the underlying treatment target was defined as human skin (*ε*_r_ = 15). A finite element method was employed for grid partitioning. Within the effective discharge region, the mesh size was set to 2 µm, with the edges of the microstructured electrodes further refined to a mesh size of 0.5 µm. In non‐plasma discharge zones, a coarser mesh size of 30 µm was utilized. In this model, air was simplified as a gas mixture comprising 79% N_2_ and 21% O_2_. The initial electron density and mean electron energy were set to 10^6^ m^−3^ and 2 eV, respectively. The specific boundary conditions for the plasma model were summarized in Table S4.

### 4.2 Construction and Driving of the Air‐Flexible CAP System

PDMS monomer and curing agent (Sylgard 184, Dow Corning, USA) were mixed at a mass ratio of 10:1. After thorough stirring, the mixture was degassed under vacuum to remove microbubbles and cast into stainless‐steel molds for curing (110°C, 30 min) [87], yielding PDMS films of various thicknesses and surface micro/nanostructures. The cured PDMS films were conformally laminated with conductive hydrogel, and carbon fiber wires were embedded as flexible electrode leads. The hydrogel used was a commercial medical electrode conductive hydrogel (Q1.0G‐80‐GMD, Dongguan Quanding Medical Supplies Co., Ltd., China). It belongs to a standard polymer electrode gel system and primarily relies on ionic conduction. Its water content was maintained at 30%–50%, and its impedance was ≤1000 Ω/cm, which ensures excellent conformal adhesion while enabling efficient charge transfer. These were connected to a custom‐designed compact high‐frequency power supply. The power supply employed a zero‐voltage switching (ZVS) topology for DC‐AC conversion, coupled to a high‐frequency step‐up transformer, enabling continuous tuning of the output peak voltage from 2.3 to 4.1 kV at a fixed driving frequency of 20 kHz. After circuit integration and structural optimization, the system was encapsulated in a 3D‐printed housing (7.2 cm× 6.0 cm× 2.0 cm; effective internal space 6.9 cm× 5.7 cm× 1.7 cm), providing both high‐voltage output capacity and portability, and tailored to meet the low‐power discharge requirements of flexible electrodes.

### 4.3 Construction of the Multi‐Parameter Discharge Diagnostic Platform

The physical characteristics of the discharge were synchronously monitored using a multi‐parameter plasma diagnostic platform, which integrated:

(1) Electrical measurements: A high‐voltage probe (P6015A, Tektronix, USA) and a current transformer (6595, Pearson, USA) were connected to an oscilloscope to acquire voltage‐current waveforms. Instantaneous power was calculated using Lissajous figures [88].

(2) Optical diagnostics: A high‐sensitivity digital camera (EOS 650D, Canon, Japan) was used to record the overall discharge morphology of AFPP at different voltages (ISO 3200, exposure time 1/10 s, aperture f/5.6). Emission spectra of reactive species were analyzed using a spectrometer (Omni‐λ750i, standard resolution 0.03 nm, Zolix, China). The characteristic emission peaks were compared against the NIST Atomic Spectra Database [89] to accurately identify the key reactive oxygen and nitrogen species.

(3) Thermal imaging: A handheld thermal camera (Uti260B, Uni‐Trend Technology Co., Ltd., China) was employed to measure electrode surface temperature fields and biointerface heat transfer. The system had a resolution of 256 × 192 pixels, a temperature accuracy of ±2°C, and a thermal sensitivity below 50 mK.

### 4.4 Mechanical Performance Testing of AFPP

The tensile mechanical properties of the flexible electrodes were evaluated at room temperature using a universal testing machine (5982, Instron, USA). Samples were prepared to dimensions of 60 mm (length) × 12 mm (width) × 2 mm (thickness) in accordance with ASTM D412 Type C. Tests included:

(1) Uniaxial tensile test: Conducted at a strain rate of 20 mm/min, with recording of maximum fracture strain and tensile strength.

(2) Cyclic tensile test: Ten stretch‐release cycles were performed at 80% strain to monitor stress‐strain hysteresis curves. At least three independent samples were tested for each group to ensure reproducibility.

(3) Deformation morphology: Photographic recording of the patch morphology under various strains and bending angles (60°, 90°, 180°).

Stress (σ) and strain (ε) were calculated using the following formulas:

(3) σ=F/A

where *F* was the applied load (N), and *A* was the initial cross‐sectional area (12 mm × 2 mm = 24 mm^2^).

(4) $$\varepsilon =\Delta L/L_{0}\;\times 100\%$$

where Δ*L* was the elongation (mm), and *L_0_* was the initial length (mm).

### 4.5 Electrical Stability Evaluation

Capacitance and impedance of the patch before and after stretching, as well as before and after continuous discharge, were measured using an LCR meter (3532‐50, HIOKI, Japan).

### 4.6 Surface Morphology Stability Evaluation

Surface morphology before and after mechanical deformation was analyzed using scanning electron microscopy (SU8600, Hitachi, Japan). Samples were mounted on copper stubs and sputter‐coated with a 5 nm Au/Pd layer for 60 s using an ion sputter coater (MC100, Hitachi, Japan) to enhance conductivity. SEM imaging was performed at an accelerating voltage of 5 kV and a working distance of 8 mm. Surface features such as cracks, pores, and delamination were comparatively assessed. To analyze the surface chemical states of the PDMS, X‐ray photoelectron spectroscopy (XPS, K‐Alpha, Thermo Scientific, USA) was performed. A monochromatic Al Kα X‐ray source (1486.6 eV) was utilized, and the analysis spot size was set to 400 µm in diameter. Both survey scans and high‐resolution spectra for C 1s and Si 2p were acquired. The evolution of surface chemical bonds was subsequently assessed via binding energy calibration and peak fitting of the characteristic peaks.

### 4.7 Detection of Gaseous Reactive Species

Under steady discharge conditions, the concentrations of nitrogen oxides (NO, NO_2_) were measured using a handheld gas analyzer (Optima, MRU, Germany). The testing was performed at a peak voltage of 3.5 kV for a discharge duration of 6 min, with instantaneous concentration data collected simultaneously at distances of 1–4 cm from the discharge region. O_3_ concentrations were measured with a portable ozone detector (GT1000‐03‐LH, Koron, China) under identical conditions. Upon startup, these instruments automatically complete calibration, and the stable values displayed during the measurement process were directly recorded as the final results. The detectors operate based on an electrochemical sensing principle: once the target gas diffuses into the sensor, corresponding redox reactions occur on the surface of the working electrode. This generates a current signal that was directly proportional to the gas concentration, thereby enabling quantitative gas analysis. All gaseous measurements were conducted with three parallel replicates to minimize experimental error and improve data reliability.

### 4.8 Quantification of Liquid‐Phase Reactive Species

A barrier model was established by covering a 30 mL receiving chamber with chicken skin (subcutaneous fat layer removed) to serve as a barrier layer, simulating the barrier effect of biological skin. The AFPP was applied to the outer surface of the chicken skin and operated at 2.9 kV (6 min) and 3.5 kV (30 s, 3 min, and 6 min). For each condition, three independent samples were tested for statistical analysis. Following treatment, the receiving liquid beneath the barrier was immediately collected, and the RONS was quantified to characterize the cumulative levels of reactive species generated by the AFPP that permeated through the skin barrier.

Among the ROS, H_2_O_2_ was quantified using a hydrogen peroxide assay kit (S0038, Beyotime, China). Its method relies on the oxidation of Fe^2+^ to Fe^3+^ by H_2_O_2_, which subsequently forms a purple complex with xylenol orange, enabling the determination of the hydrogen peroxide concentration. In the experiment, a 50 µL sample was reacted with 100 µL of the reagent in the dark at room temperature (15∼30°C) for 30 min, and the absorbance was then recorded at 560 nm. O_3_ was measured with an ozone assay kit (090012, HKM, China), where 15 mL of the sample was reacted with the reagent for 3 min. The mixture was then transferred to a 96‐well plate, and the colorimetric signal generated by the oxidation of I^−^ by O_3_ was measured at 525 nm [90]. ·OH was detected using terephthalic acid (TA, P816020‐100 g, Macklin, China) as a specific probe. Under alkaline conditions, TA reacts with ·OH to form highly fluorescent 2‐hydroxyterephthalic acid (HTA). Its concentration can be characterized by measuring the fluorescence intensity at an excitation wavelength of 315 nm and an emission wavelength of 425 nm. O_2_^−^ was quantified with a superoxide anion assay kit (R30343‐50T, OriLeaf, China). O_2_^−^ reacts with hydroxylamine to generate NO_2_^−^, which further reacts with sulfanilic acid and naphthylamine to produce a pink azo compound. Quantitative analysis was achieved by measuring the absorbance at 530 nm [91].

As for RNS, NO_2_^−^ was measured using the Griess reagent (S0021, Beyotime, China). A 50 µL sample was sequentially mixed with Griess reagents I and II to undergo a diazotization reaction, yielding a red azo compound with a characteristic absorption peak at 520–540 nm. NO_3_^−^ was determined by reacting the sample with a 450 µmol/L sodium hydroxide solution and measuring the UV absorbance at 220 nm. ONOO^−^ was quantified using a peroxynitrite anion assay kit (BB‐46155‐100T, BestBio, China). The fluorescent probe O56 in the kit was oxidized in the presence of ONOO^−^ to produce a green fluorescent product, the fluorescence intensity of which was positively correlated with the ONOO^−^ content in the sample. A 100 µL sample was mixed with 10 µL of the diluted O56 probe working solution, incubated in the dark at 37°C for 15–30 min, and the fluorescence signal was recorded at an excitation wavelength of 488 nm and an emission wavelength of 525 nm.

All measurements were performed using a multifunctional microplate reader or a fluorescence spectrophotometer. Each condition was tested in parallel replicates to ensure accuracy and reproducibility.

### 4.9 Detection of Ultraviolet Exposure

To evaluate the radiation safety of the AFPP during operation, we measured its UV radiation exposure under the established maximum treatment dose condition (3.5 kV‐6 min). A UV irradiance meter (LS135, Linshang, China) equipped with dedicated probes for the 230–400 nm band was employed to monitor the cumulative radiation energy in real time, with a measurement accuracy of ±5%. During the test, the UV detector probe was placed in direct contact with and squarely facing the effective discharge area of the AFPP, simulating the maximum possible radiation dose received by the tissue surface during in vivo treatment. Throughout the test, the instrument directly and accurately recorded the cumulative energy exposure values for each band (µJ/cm^2^ or mJ/cm^2^). Data were recorded at 1 min intervals, and all measurements were repeated three times under ambient air conditions to ensure the reliability and reproducibility of the data.

### 4.10 Animal Experiments

Female BALB/c nude mice (4‐5 weeks old, 18–20 g) were purchased from Charles River Laboratory Animal Technology Co., Ltd. (Beijing, China). A total of 30 mice were used in the in vivo study, divided into 5 groups (n = 6 per group). 1 × 10^6^ B16‐F10 melanoma cells were injected subcutaneously into each mouse. When the average tumor volume reached 40 mm^3^, the mice were randomly allocated into experimental groups and subjected to daily AFPP treatment from day 7 to day 14. Tumor dimensions were measured with digital calipers, and volumes were calculated using the formula:

(5) $$V=\pi /6\;\times \;L\;\times \;W^{2}$$

where *L* was the longest diameter and *W* was the shortest diameter. Body weights were recorded once daily from day 6 (one day before the first AFPP treatment) until the end of the experiment to evaluate systemic tolerance. At the end of the treatment period, mice were euthanized, and tumors were excised, photographed, and weighed. Tumor size measurements and hematological tests were performed on all mice. Among them, 3 mice were subjected to proteomics and Western blot, and the other 3 were used for IHC and H&E staining. Power analysis using G*power confirmed that the statistical power was above 0.8, supporting the validity of the conclusions. All animal procedures were conducted at the Peking University Health Science Center and approved by the Peking University Animal Care and Use Committee. The associated ethical approval number was: DLASBE0765.

### 4.11 Tissue Protein Extraction

Total protein from melanoma tissues was extracted using a total protein extraction kit for animal tissues (Solarbio, China). Fresh tumor tissue (15–20 mg) was placed in a pre‐cooled centrifuge column tube and homogenized 50–60 times with the provided pestle to disperse the sample. A total of 200 µL lysis buffer containing protease inhibitors was added in two steps, followed by further grinding (30–60 times) until homogenized. After standing at room temperature for 1–2 min, the lysate was centrifuged at 14,000–16,000 ×g for 1–2 min, and the supernatant was collected as the total protein solution, which was either used immediately for analysis or stored at −80°C.

### 4.12 Western Blot Analysis

Protein samples were mixed with 5× loading buffer at a ratio of 4:1 and denatured at 100°C for 10 min. Equal amounts of protein (20–30 µg per lane) together with pre‐stained molecular weight markers were separated on 10% SDS‐PAGE gels (80 V for stacking gel, 100 V for separating gel, 90 min). Proteins were transferred to PVDF membranes by wet transfer (300 mA, 90 min). Membranes were blocked with 5% skim milk at room temperature for 1 h, followed by incubation with primary antibodies (1:1000 dilution) overnight at 4°C. After TBST washes, membranes were incubated with HRP‐conjugated secondary antibodies (1:3000 dilution) for 1 h at room temperature. Protein bands were visualized by ECL detection using a chemiluminescence imaging system.

### 4.13 Hematoxylin‐Eosin (H&E) Staining

Paraffin sections of tumor tissues were deparaffinized with xylene (2 × 10 min), rehydrated through graded ethanol (100% × 2 × 5 min, 90% × 5 min, 75% × 5 min), and rinsed in running water. Frozen sections were warmed at −20°C for 5–10 min and washed after fixation if necessary. Sections were stained with 0.5% hematoxylin for 3–5 min, rinsed in running water, differentiated for 2–5 s, blued, and dehydrated through 85% and 95% ethanol (5 min each). Slides were then counterstained with 0.05% eosin for 5 min, dehydrated in absolute ethanol, cleared in xylene, and mounted with neutral resin. After staining, nuclei appeared blue‐purple and cytoplasm red, allowing for morphological evaluation.

### 4.14 Immunohistochemistry (IHC)

Immunohistochemistry was performed using a DAB detection system (Servicebio, China) to assess protein distribution in tumor tissues. After deparaffinization and rehydration, antigen retrieval was carried out in sodium citrate buffer (pH 6.0) by microwave heating. Endogenous peroxidase activity was blocked with 3% H_2_O_2_ at room temperature. After blocking with 5% goat serum, sections were incubated overnight at 4°C with primary antibodies (e.g., PDK1). After TBST washing, sections were incubated with HRP‐conjugated secondary antibodies for 30 min at 37°C, followed by DAB chromogenic development for 5∼10 min and hematoxylin counterstaining for 1 min. Images were acquired using a panoramic digital slide scanner (200× magnification) and subjected to quantitative analysis.

### 4.15 Proteomic Analysis

Melanoma tissues were rinsed with PBS to remove debris and blood, snap‐frozen in liquid nitrogen for 3–4 h, stored at ‐80°C, and transported on dry ice to Qingke Biotechnology Co., Ltd. (China) for data‐independent acquisition (DIA) proteomic analysis. Samples were ground in liquid nitrogen and lysed in buffer containing 8 M urea, 1 mM PMSF, and 2 mM EDTA, followed by sonication on ice for 5 min. The lysates were centrifuged at 15,000 ×g for 10 min at 4°C, and the supernatants were collected. Protein concentrations were determined by the BCA method. For each sample, 100 µg of protein was diluted to 200 µL, reduced with DTT (5 mM, 37°C, 45 min), alkylated with IAA (11 mM, room temperature, dark, 15 min), and digested overnight with trypsin at 37°C in 25 mM ammonium bicarbonate. The reaction was quenched by adjusting the pH to 2–3 with TFA, and peptides were desalted using C18 cartridges and quantified. Samples were separated using a Vanquish Neo UHPLC nano‑liquid chromatography system (Thermo Scientific, USA). Mobile phase A consisted of 0.1% formic acid in water, and mobile phase B was 0.1% formic acid in 100% acetonitrile. Separation was performed in a trap‑and‑elute dual‑column configuration: the trap column was a PepMap Neo Trap Cartridge (300 µm × 5 mm, 5 µm), and the analytical column was an Easy‑Spray PepMap Neo UHPLC column (150 µm × 15 cm, 2 µm). The analytical column temperature was maintained at 55°C by an integrated column oven. The sample injection amount was 200 ng, with a flow rate of 2.5 µL/min. The effective separation gradient duration was 6.9 min, and the total instrument run time was 8 min per sample. The samples were then analyzed using an Orbitrap Astral mass spectrometer. Protein identification was performed with DIA‐NN (v1.8.1), and quantification was conducted using MaxLFQ. Functional annotation, differential protein screening, and enrichment analyses were performed using GO and KEGG databases.

### 4.16 Hematological Analysis

Approximately 200 µL of blood was collected from the orbital venous plexus of melanoma‐bearing mice into EDTA anticoagulant tubes and gently mixed 8–10 times. WBC, platelet, RBC and Hb levels were analyzed using an automated hematology analyzer (XS‐800i, Sysmex, Japan) to evaluate the impact of tumors on the hematopoietic system.

### 4.17 RNA Extraction and RT‐qPCR Analysis

Total RNA from tumor tissues was extracted using the AipPure RNA extraction kit (Aibosi, China) according to the manufacturer's instructions. Briefly, tissues were lysed in RL1 buffer, digested with Proteinase K, and subjected to genomic DNA removal, isopropanol precipitation, and column purification. Purified RNA was stored at ‐80°C. For reverse transcription, ≤2 µg of RNA was converted into cDNA using UltraScript RT MasterMix with gDNA Remover (42°C for 15 min, 85°C for 5 s). qPCR was performed using the SYBR Green method in 20 µL reactions containing ≈400 ng cDNA template and 0.4 µM specific primers. Amplification conditions were: 95°C for 30 s, followed by 40 cycles of 95°C for 5 s, 60°C for 20 s, and 72°C for 25 s. A melting curve analysis was conducted to verify amplification specificity. Relative gene expression was calculated using the 2‐ΔΔCt method, normalized to internal reference genes, and compared with the control group.

## Author Contributions

Methodology: **L.‐X.Z**., **A.T**., **H.‐S.Y**.; Formal Analysis: **L.‐X.Z**., **A.T**., **H.‐S.Y**., **S.X**., **A.Y**.; Data Curation: **L.‐X.Z**., **A.T**., **H.‐S.Y**., **A.Y**., **M.‐Z.W**. Visualization: **L.‐X.Z**., **H.‐S.Y**.; Writing – original draft: **L.‐X.Z**.; Writing‐revisions, **W.‐L.S**., **L.‐X.Z**., **H.‐S.Y**., **S.X**., **W.‐Z. Si**.; Writing – review and editing: all authors; Funding acquisition: **W.‐Z. Si**., **R.‐X.W**.; Supervision and Conceptualization: **W.‐Z. Si**., **R.‐X.W**. All authors read and approved the final manuscript.

## Conflicts of Interest

The authors declare no conflict of interest.

## Supporting information




**Supporting File 1**: advs76049‐sup‐0001‐SuppMat.docx.

**Supporting File 2**: advs76049‐sup‐0002‐MovieS1.mp4.

**Supporting File 3**: advs76049‐sup‐0003‐MovieS2.mp4.

**Supporting File 4**: advs76049‐sup‐0004‐MovieS3.mp4.

**Supporting File 5**: advs76049‐sup‐0005‐MovieS4.mp4.

## Data Availability

The data that support the findings of this study are available from the corresponding author upon reasonable request.
