# Supplementary material for: All‐Flex Plasma Patch for In Vivo Delivery of Reactive Species
Source: Adv Sci (Weinh). 2026 Jun 11;13(45):e76049. doi: 10.1002/advs.76049 (PMC13336424; doi:10.1002/advs.76049)
Supplement: Supplementary file 1 [file ADVS-13-e76049-s003.docx]

Supporting Information for

**All-flex plasma patch for *in vivo* delivery of reactive species**

**Luxiang Zhao^1,†^, Abulaihaiti Tuergong^2,†^, Hanshu Yang^1^, Shuang Xue^1^, An Yan^1^, Mingzhen Wang^2^, Weilong Song^2^, Wenzhe Si^2,^*, Ruixue Wang^1,^***

^1^ College of Mechanical and Electrical Engineering, Beijing University of Chemical Technology, Chaoyang District, Beijing 100029, China.

^2^ Department of Laboratory Medicine, State Key Laboratory of Vascular Homeostasis and Remodeling, Key Laboratory of Cardiovascular Molecular Biology and Regulatory Peptides, National Health Commission, Peking University Third Hospital, Beijing 100191, China.

^*^ Corresponding authors. Email: wrx@mail.buct.edu.cn (R.-X.W.);

[wenzhesi@bjmu.edu.cn](mailto:wenzhesi@bjmu.edu.cn) (W.-Z.Si.)

^†^ These two authors contributed equally to this work.

**This file includes:**

Tables S1 to S4

Legends for movies S1 to S4

Supplementary Text S1

Figures S1 to S6

**Supplementary Table S1**

**Table S1. Selection criteria for microstructures based on multi-parameter analysis.**

V_0_ represents the discharge space volume of the reference rectangular microstructure.

| Microstructure | Discharge space volume | *E*  (Along the electric field line) | *E*  (Material Surface) | Maximum electric field intensity | Support structure of AFPP |
| --- | --- | --- | --- | --- | --- |
| Rectangle | V_0_ | Uniform (Intense discharge) | Uniform (Without diffusion) | Weak | Large contact area of support structure |
| Triangle | 0.5 V_0_ | Monotonically decreasing | Nonuniform | Strong | Full surface treatment |
| Semicircle | 0.785 V_0_ | Monotonically decreasing | Nonuniform | Strong | Full surface treatment |
| Cusp | 0.215 V_0_ | Monotonically decreasing | Extremely nonuniform (Prone to local breakdown) | Extremely strong (Prone to local breakdown) | Full surface treatment |

**Text S1**

For H_2_O_2_, its major formation pathway is as follows^[1]^:

$2\text{OH}\to\text{H}_{2}\text{O}_{2}$ (S1)

The generation of ·OH primarily depends on the electrolysis of water and the reaction between excited oxygen molecules and water^[2,3]^:

$e+\text{H}_{2}\text{O}\to\text{H}+\text{OH}+e$ (S2)

$e+\text{H}_{2}\text{O}\to\text{OH}+\text{H}^{-}$ (S3)

$\text{O}\left(^{1}\text{D}\right)+\text{H}_{2}\text{O}\to2\text{OH}$ (S4)

Therefore, an increase in the water content of the background gas environment leads to a significant elevation in H_2_O_2_ concentration. In plasma discharges, O_3_ is mainly produced through the three-body recombination of oxygen atoms with oxygen molecules:

$\text{O}+\text{O}_{2}+\text{O}_{2}\to\text{O}_{3}+\text{O}_{2}$ (S5)

$\text{O}+\text{O}_{2}+\text{O}_{3}\to\text{O}_{3}+\text{O}_{3}$ (S6)

The abundant reactive oxygen species generated during discharge thus play a central role in the production of both O_3_ and H_2_O_2_. For NO, its generation in plasma is primarily attributed to the combination of N and O atoms, as well as excited NO molecules^[4-6]^:

$\text{O}+\text{N}+\text{N}_{2}\to\text{NO}+\text{N}_{2}$ (S7)

$\text{O}+\text{N}+\text{O}_{2}\to\text{NO}+\text{O}_{2}$ (S8)

$\text{O}+\text{N}_{2}\to\text{NO}+\text{N}$ (S9)

$\text{O}_{2}+\text{N}\to\text{NO}+\text{O}$ (S10)

Accordingly, elevated levels of nitrogen and oxygen atoms and molecules also contribute positively to the increase in NO concentration.

**Figure S1.**

**Figure S1. Lissajous figures of AFPP under different driving voltages.**

**Representative voltage-charge (*U*-*U*c) curves obtained at 2.3 kV, 2.9 kV, 3.5 kV, and 4.1 kV.**

**Supplementary Table S2**

**Table S2. Capacitance variation (*C*) of AFPP and its constituent materials before and after 10 cycles of loading-unloading. AFPP (whole device) refers to the integrated multilayer structure (PDMS-hydrogel-PDMS); PDMS with microgrooved structure and PDMS correspond to the single-layer dielectric substrates used for comparison.**

| **Sample type** | ***C*_0_ (pF)** | ***C*_1_ (pF)** | **Δ*C* (pF)** | **Δ*C*/*C*_0_ %** |
| --- | --- | --- | --- | --- |
| **AFPP (whole device)** | 5.858 | 5.768 | 0.09 | 1.536% |
| **PDMS with microgrooved structure** | 7.179 | 7.296 | 0.117 | 1.629% |
| **PDMS** | 7.488 | 7.564 | 0.076 | 1.015% |

**Supplementary Table S3**

**Table S3. Capacitance variation (*C*) of AFPP and its constituent materials before and after 720 min of discharge. *AFPP (whole device)* refers to the integrated multilayer structure (PDMS-hydrogel-PDMS); *PDMS with microgrooved structure* and *PDMS* correspond to the single-layer dielectric substrates used for comparison.**

| **Sample type** | ***C*_0_ (pF)** | ***C*_1_ (pF)** | **Δ*C* (pF)** | **Δ*C*/*C*_0_ %** |
| --- | --- | --- | --- | --- |
| **AFPP (whole device)** | 8.977 | 8.945 | 0.032 | 0.356% |
| **PDMS with microgrooved structure** | 11.050 | 11.108 | 0.058 | 0.525% |
| **PDMS** | 10.789 | 10.814 | 0.025 | 0.232% |

**Figure S2.**

**Figure S2. SEM images and elemental mappings (Si, C, and O) of the AFPP surface before and after different discharge durations.**

**Figure S3.**

**Figure S3. XPS analysis of the surface after 720 min of discharge. A) XPS survey spectrum and high-resolution C 1s and Si 2p spectra of the untreated (0 min) sample. B) XPS survey spectrum and high-resolution C 1s and Si 2p spectra of the sample following 720 min of discharge.**

**Figure S4.**

**Figure S4. Body weight monitoring during treatment.**

**Body weights of mice in all treatment groups remained stable throughout therapeutic period, with no statistically significant intergroup differences (n = 6 per group, mean ± SD).**

**Figure S5.**

**Figure S5. Molecular mechanisms underlying AFPP-mediated melanoma therapy. A) Volcano plot of differentially expressed proteins between the 3.5 kV-6 min group and control. B) GO**

**enrichment analysis of biological processes in the 3.5 kV-6 min group. C) KEGG pathway analysis of signaling and metabolic pathways in the 3.5 kV-6 min group.**

**Figure S6.**

**Figure S6. Schematic diagram of the simulation of the AFPP discharge device with a semicircular microstructure.**

**Supplementary Table S4**

Table S4. Boundary conditions used for the AFPP model in COMSOL simulations.

| **Boundary** | **Electrostatic condition** | **Species condition** |
| --- | --- | --- |
| AB | Electric potential | NA |
|  | *V*=3.5 kV |  |
| AC/BH | *E_r_*=0 | $\frac{\partial n_{i}}{\partial r}=0$ |
| CH | Electric potential | Continuity $n\cdot\overrightarrow{\vec{\Gamma}_{i}}=0$  |
|  | *V*=0 kV |  |
| CD/DE/EF/FG/GH  CI/ID/DJ/JE/EK  KF/FL/LG/GM/MH | Field continuity $-n\cdot\left(\overrightarrow{D_{1}}-\overrightarrow{D_{2}}\right)=\rho_{\text{s}}$  | Wall loss |

**Supplementary references**

[1] Y. Hong, B. Dail, A. Sternin, O. Haidn, 2018 Joint Propulsion Conference, Cincinnati, Ohio, **2018**, 9, July.
[2] W.G. Mallard, F. Westley, J.T. Herron, R.F. Hampson, D.H. Frizzell, Washington, DC, USA: National Institute of Standards and Technology, **1992**.
[3] Y. Itikawa, N. Mason, J. Phys. Chem. Ref. Data **2005**, 34, 1.
[4] I. A. Kossyi, A. Y. Kostinsky, A. A. Matveyev, V. P. Silakov, Plasma Sources Sci. Technol. **1992**, 1, 207.
[5] F. J. Gordillo-V´azquez, J. Phys. D: Appl. Phys. **2008**, 41, 234016.
[6] O. Eichwald, M. Yousfi, A. Hennad, M. D. Benabdessadok, J. Appl. Phys. **1997**, 82, 4781.

**Supplementary Movies**

**Movie S1. Simulated electron density distribution of AFPP with microgrooved PDMS substrate. The movie shows the time‐resolved evolution of discharge within microgrooved structures, visualized as the logarithm of electron density (log₁₀(*n*ₑ)).**

**Movie S2. Manual mechanical deformation of the AFPP. This video shows the AFPP undergoing manual bending, stretching, twisting, and elastic recovery, demonstrating its high flexibility and mechanical robustness.**

**Movie S3. *In vivo* therapeutic application of AFPP at 2.9 kV. The patch was conformally attached to the tumor site of melanoma-bearing mice prior to plasma discharge.**

**Movie S4. *In vivo* therapeutic application of AFPP at 3.5 kV. The patch was conformally attached to the tumor site of melanoma-bearing mice prior to plasma discharge.**
